# Robust and Reversible Supramolecular Adhesive via Dynamic Covalent Bond Crosslinking‐Induced Assembly of Metal‐Coordinated Nanoparticles

**DOI:** 10.1002/advs.202505122

**Published:** 2025-07-30

**Authors:** Yanyan Guo, Mengran Zhang, Shuanggen Wu, Xunqiu Wang

**Affiliations:** ^1^ State Key Laboratory of Coking Coal Resources Green Exploitation School of Chemical Engineering Zhengzhou University Zhengzhou 450001 P. R. China

**Keywords:** metal‐coordinated nanoparticles, reversible adhesion, supramolecular polymer networks

## Abstract

Supramolecular adhesives, praised for their stimuli‐responsiveness and reversibility, have gained significant attention. However, most of these adhesives demonstrate low tolerance to extreme environments and exhibit diminished adhesion performance after cyclic adhesion testing. Herein, a hierarchical self‐assembly strategy is introduced for the construction of supramolecular polymer networks (poly(UIO‐TA)) through dynamic disulfide bond crosslinking‐induced assembly of metal‐coordinated nanoparticles (UIO‐TA). The UIO‐TA were synthesized via a stepwise coordination‐driven assembly process, wherein the zirconium ion coordinated with terephthalic acid and thioctic acid. Furthermore, these UIO‐TA were crosslinked through ring‐opening polymerization of dynamic disulfide bonds, resulting in the formation of poly(UIO‐TA). Poly(UIO‐TA) exhibited tough, durable, and reversible adhesion on diverse substrates, maintaining its effectiveness under mild to harsh conditions, including high temperatures (120 °C), organic solvents (e.g., dimethyl sulfoxide, ethanol), and strong acids (e.g., sulfuric acid). The adhesion performance of poly(UIO‐TA) is superior to poly(thioctic acid) (poly(TA)) and most reported supramolecular adhesives. These attributes can be ascribed to the synergistic interplay of metal coordination bonds, hydrogen bonds, and dynamic disulfide bonds. Notably, after the five‐cycle adhesion tests, the adhesion strength of poly(UIO‐TA) increased by ≈2.5 times, which can primarily be attributed to increased cohesion energy resulting from the further ring‐opening polymerization of disulfide bonds.

## Graphical abstract

A supramolecular polymer network is constructed through the assembly of metal‐coordinated nanoparticles induced by the crosslinking of dynamic covalent disulfide bond. The resulting supramolecular adhesive exhibited excellent adhesion performance, including strong adhesion strength, exceptional solvent resistance, and distinct reversible adhesion.

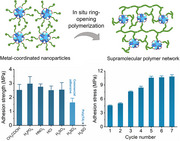

## 1 Introduction

Adhesion is a ubiquitous phenomenon in nature, as exemplified by mussels to wet surfaces and geckos scaling vertical walls.^[^ ^1^, ^2^ ^]^ Drawing inspiration from these natural processes, reversible and directional noncovalent interactions—such as hydrogen bonding, host–guest interactions, and metal‐ligand coordination—offer valuable insights for designing supramolecular adhesives.^[^ ^3^, ^4^, ^5^, ^6^, ^7^ ^]^ Compared to conventional polymeric adhesives, supramolecular adhesives exhibit exceptional properties, including stimuli‐responsiveness, reversibility, and recyclability.^[^ ^8^, ^9^, ^10^, ^11^, ^12^, ^13^ ^]^ As a result, they have gained significant attention across fields, including biomedicine, wearable devices, and soft actuators.^[^ ^14^, ^15^, ^16^, ^17^ ^]^ However, most of supramolecular adhesives exhibit inadequate adhesion strength and suboptimal durability,^[^ ^18^, ^19^, ^20^, ^21^, ^22^, ^23^ ^]^ particularly under harsh conditions, which significantly hinders their prolonged use in environments with high humidity, water, organic solvents, acidic solutions, or extreme temperatures.^[^ ^24^, ^25^, ^26^, ^27^ ^]^ Consequently, it is crucial to develop advanced supramolecular adhesives that demonstrate enhanced resistance to a variety of challenging environmental factors.

In addition, a significant challenge associated with supramolecular adhesives is the trade‐off between adhesion strength and reversibility. To address this challenge, incorporating dynamic covalent bonds into noncovalent crosslink networks offers a promising strategy to enhance both the adhesion strength and the dynamic, reversible properties of supramolecular adhesives.^[^ ^28^, ^29^, ^30^, ^31^, ^32^, ^33^ ^]^ Dynamic covalent crosslinks—such as disulfide bridges, Diels–Alder linkages, boron ester bonds, and imine bonds—offer strength and stability comparable to conventional covalent bonds in the absence of external stimuli.^[^ ^34^, ^35^ ^]^ The dynamic covalent network significantly contributes to the cohesive energy of the adhesive, preventing deformation under mechanical stress. Furthermore, dynamic covalent and noncovalent crosslink networks can be engineered to respond to specific stimuli (e.g., light, temperature, or redox conditions), enabling efficient topological rearrangements.^[^ ^36^, ^37^, ^38^ ^]^ This facilitates the in situ formation of an adhesive layer, enhancing molecular‐interface interactions and increasing the effective adhesive surface area on suboptimal substrates, ultimately improving interfacial adhesion.^[^ ^39^, ^40^, ^41^ ^]^ While the adhesive strength typically remains constant or even decreases as the cycle number increases, there are few reports indicating that the adhesive strength improves with increasing cycles.

**Scheme 1:**
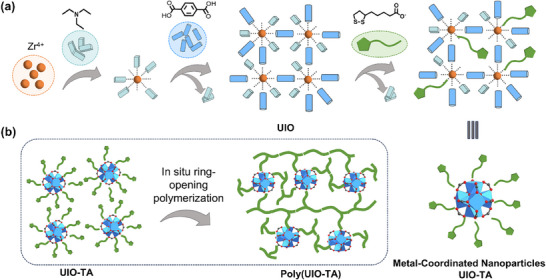
Construction of SPNs via dynamic covalent bond crosslinking‐induced assembly of UIO‐TA.

In this study, we present a hierarchical self‐assembly (HAS) strategy for constructing supramolecular polymer networks (SPNs) through the dynamic covalent bond crosslinking‐induced assembly of metal‐coordinated nanoparticles (UIO‐TA) (**Scheme** **1**). The nanoparticles were synthesized via a stepwise coordination‐driven assembly, where the zirconium ion (Zr^4+^) coordinated with terephthalic acid (BDC) and thioctic acid (TA). These UIO‐TA were used as building blocks and were in situ crosslinked through ring‐opening polymerization of dynamic disulfide bonds and coordination bonds via a solvent evaporation method. The resulting supramolecular polymer (poly(UIO‐TA)) exhibits a range of distinct physicochemical properties, including high hardness, excellent thermal stability, and remarkable solvent resistance, attributed to the synergistic interplay of coordination bonds, hydrogen bonding interactions, and dynamic disulfide bonds. Additionally, the reversible nature of these bonds imparts significant temperature‐responsive viscoelasticity to the polymer. This material demonstrates excellent potential as a hot‐melt adhesive, showing superior adhesion performance across a variety of substrates under both mild and harsh conditions, including extreme temperatures (−20 to 120 °C), exposure to seawater, polar organic solvents (e.g., ethanol and dimethyl sulfoxide (DMSO)), and immersion in strong acidic solutions (e.g., hydrochloric acid (HCl), sulfuric acid (H_2_SO_4_), and nitric acid (HNO_3_) solution). Moreover, the adhesion strength of poly(UIO‐TA) gradually increased after multiple cycling experiments.

## 2 Results and Discussion

### 2.1 Synthesis and Characterization of Poly(UIO‐TA)

**Figure 1:**
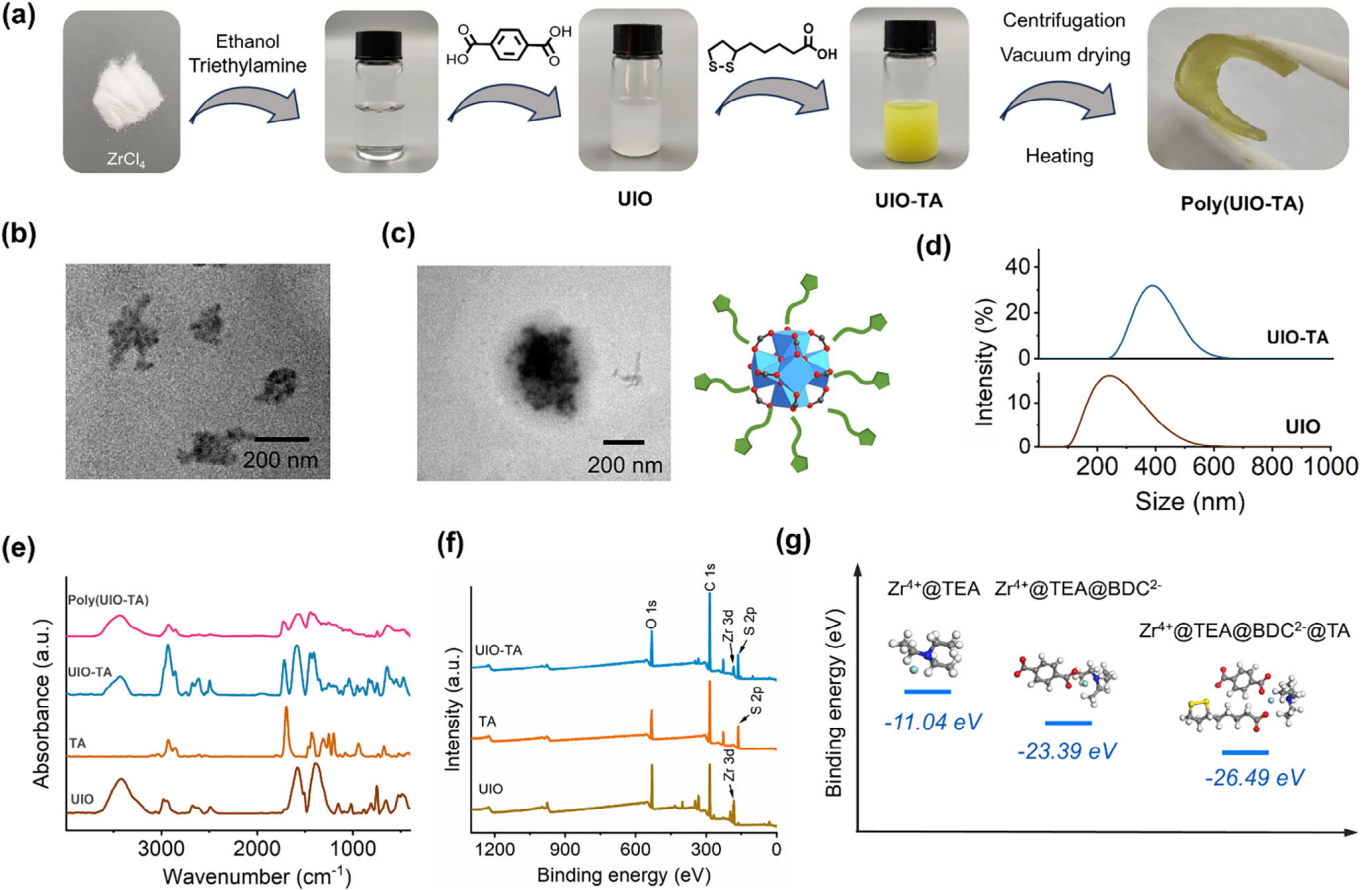
Fabrication and characterization of poly(UIO‐TA) network. a) Schematic representation of the synthesis route for poly(UIO‐TA). b) TEM image of UIO. c) TEM image of UIO‐TA. d) Size distribution of UIO‐TA and UIO, analyzed by dynamic light scattering. e) FT‐IR spectra of poly(UIO‐TA), UIO‐TA, TA, and UIO. f) XPS spectra of UIO‐TA, TA, and UIO. g) DFT calculations of binding energies for the complexes Zr^4+^@TEA, Zr^4+^@TEA@BDC^2−^, and Zr^4+^@TEA@BDC^2−^@TA.

First, UIO‐TA was fabricated through the stepwise coordination‐driven assembly of Zr^4+^ with BDC and TA (**Figure** **1**a). The morphology and chemical structure of UIO‐TA were rigorously analyzed. Transmission electron microscopy (TEM) images revealed the sizes of UIO‐TA and UIO. As shown in Figure 1b,c, UIO‐TA had a larger diameter (≈400 nm) compared to UIO (≈200 nm), confirming the successful TA coating on UIO. Dynamic light scattering analysis showed that the mean hydrodynamic diameters of UIO‐TA and UIO were ≈400 and 300 nm, respectively (Figure 1d). The polydispersity index (PDI) of UIO‐TA and UIO was 0.179 and 0.262, respectively, which indicates that they possess a narrow size distribution. Fourier transform infrared (FT‐IR) spectroscopy revealed three characteristic peaks at 474, 646, and 746 cm^−1^, which can be assigned to the HO─Zr─OH, µ_3_‐OH, and Zr─O groups, respectively. The C═O stretching vibration of the carboxyl group in TA was detected at 1689 cm^−1^, which exhibited a blue shift to 1722 cm^−1^ in the UIO‐TA, indicating the successful grafting of TA onto the UIO‐TA (Figure 1e). Time‐dependent UV–vis spectra and X‐ray photo‐electron spectroscopy (XPS) analysis of UIO‐TA further revealed that occupation of unsaturated Zr sites of UIO by the carboxyl group of TA (Figure 1f; Figures S1 and S2, Supporting Information). Additionally, positron annihilation lifetime spectroscopy showed that the defects in UIO were reduced after the addition of TA, suggesting that TA could partially eliminate open metal sites, facilitating the formation of UIO‐TA (Figure S3, Supporting Information). To investigate the coordination assembly of UIO‐TA, the intermolecular interactions between Zr^4+^ and various ligands were calculated using density functional theory (DFT). The bind energies for the complexes Zr^4+^@TEA, Zr^4+^@TEA@BDC^2−^, and Zr^4+^@TEA@BDC^2−^@TA were −11.04, −23.39, and −26.49 eV, respectively (Figure 1g). These results suggest that Zr^4+^ sites are initially coordinated with triethylamine (TEA). However, TEA can be sequentially replaced by BDC^2−^ or TA^−^ ions, promoting the progressive assembly of UIO‐TA through ligand exchange. It is inferred that TEA could promote deprotonations of the BDC and TA ligands into BDC^2−^ and TA^−^ ions. The “activated” BDC^2−^ and TA^−^ ligands can coordinate to the Zr^4+^ form metal–organic nanoparticles. Moreover, the interaction between protonated TEA molecule and Zr^4+^ is weaker than that of Zr^4+^@TEA complex.^[^ ^42^, ^43^ ^]^

**Figure 2:**
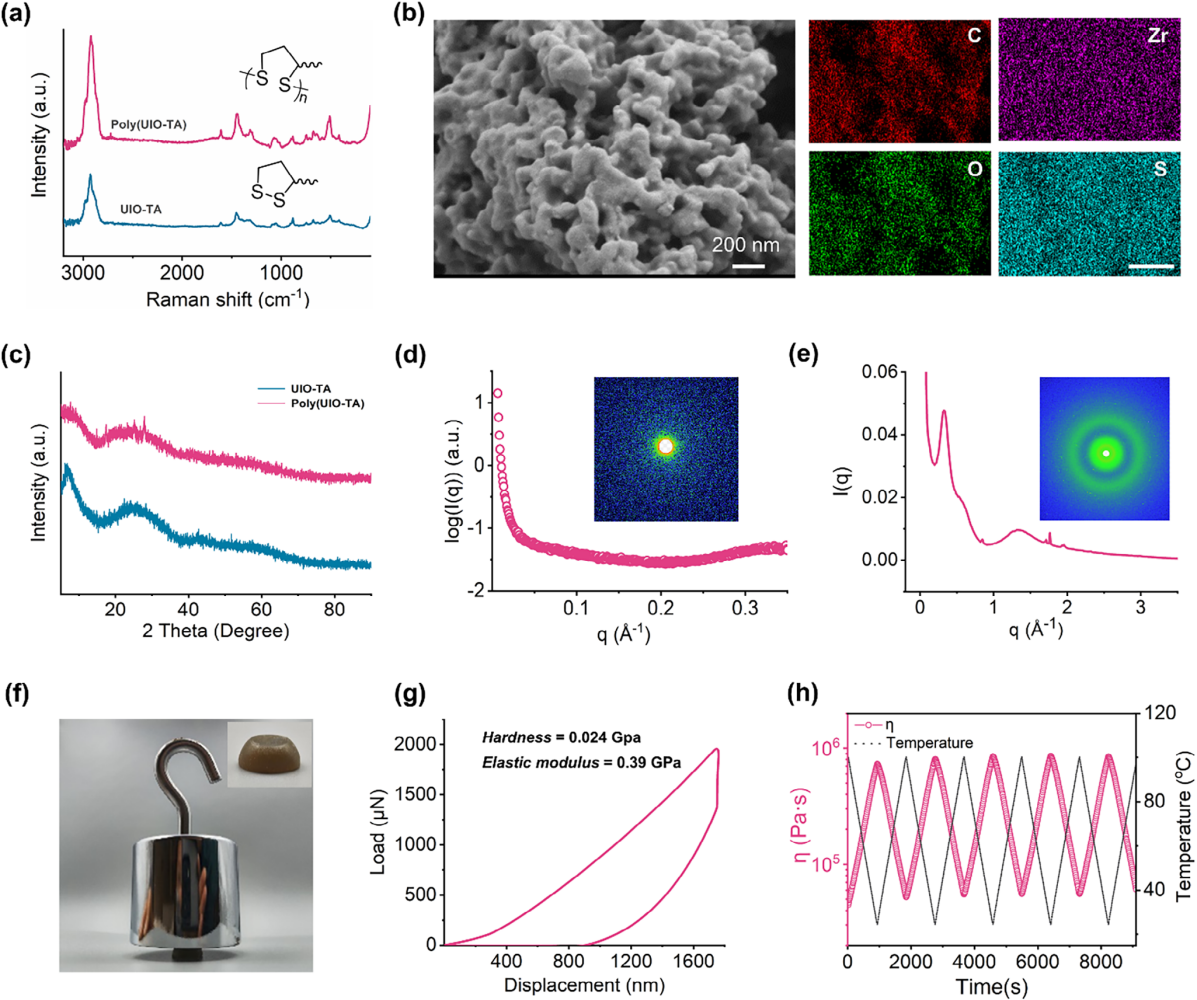
Properties of poly(UIO‐TA). a) Raman spectra of poly(UIO‐TA) and UIO‐TA. b) SEM image of poly(UIO‐TA) (left) and elemental mapping of C, O, Zr, and S. Scale bar: 5 µm (right). c) PXRD pattern of UIO‐TA and poly(UIO‐TA). d) SAXS spectra of poly(UIO‐TA). e) WAXS spectra of poly(UIO‐TA). f) Macroscopic weight‐loading tests of poly(UIO‐TA). g) Load–displacement curves of poly(UIO‐TA). h) Temperature‐dependent reversible composite viscosity of poly(UIO‐TA) composites.

The poly(UIO‐TA) was obtained by centrifuging the UIO‐TA suspension, followed by vacuum drying at 25 °C for over three days. To better understand this process, time‐dependent Raman spectra of UIO‐TA and poly(UIO‐TA) were collected (**Figure** **2**a; Figure S5, Supporting Information). The Raman peaks at 270 and 1611 cm^−1^ were attributed to the in‐plane bending of the linker to the metal clusters and the C═C stretching of the aromatic rings in BDC, respectively. Notably, the Raman peak at 507 cm^−1^ bifurcated into two peaks, providing evidence for the existence of intermolecular S─S bonding, which is facilitated by ring‐opening polymerization.^[^ ^44^ ^]^ This indicates that the crosslinking of UIO‐TA occurs through the spontaneous polymerization of the S─S bonds in TA, alongside the evaporation of ethanol and TEA. To further investigate the distribution of various components within poly(UIO‐TA), time‐of‐flight secondary ion mass spectrometry was used, revealing the spatial arrangement of ring‐opened TA fragments, aggregated S3 fragments, and Zr^4+^ ions (Figures S6–S9, Supporting Information). Energy‐dispersive X‐ray spectroscopy analysis confirmed the homogeneous distribution of carbon, oxygen, sulfur, and zirconium across the surface of the poly(UIO‐TA) (Figure 2b). The microscopic structure of poly(UIO‐TA) was analyzed using scanning electron microscopy (SEM), which showed a 3D network with an interconnected porous architecture formed through nanoparticle crosslinking (Figure 2b; Figure S10, Supporting Information).

Powder X‐ray diffraction (PXRD) data revealed no distinct diffraction peaks for UIO‐TA, indicating the absence of long‐range order (Figure 2c). This amorphous characteristic contrasts with the well‐ordered lattice structures typically seen in metal‐oxide clusters or metal–organic framework nanoparticles. It suggests that the as‐formed Zr‐clusters, which are supramolecularly grafted with multiple TA chains, experience conformationally unfavorable crystallization behaviors. As a result, upon solvent evaporation, they undergo vitrification, resulting in the formation of an amorphous bulk material. The in situ polymerization of the Zr‐clusters into crosslinked networks preserves their local coordination structures, as evidenced by the similarity between the XRD patterns of poly(UIO‐TA) and its precursors. To gain more insight into the condensed structures of poly(UIO‐TA), small‐angle and wide‐angle X‐ray scattering (SAXS and WAXS) analyses were performed (Figure 2d,e). The SAXS data, presented in a double logarithmic plot, clearly shows a typical Guinier region with a slope approaching zero in the low *Q* range (0.0004–0.002 Å^−1^), corresponding to a local inhomogeneity with a radius of gyration (*R*_g_) of 70.9 nm. This suggests that the Zr‐clusters are not uniformly distributed but tend to aggregate into high‐density domains within the poly(thioctic acid) (poly(TA)) matrix. This observation is consistent with the real‐space TEM imaging results, which confirm the validity of the SAXS data analysis. The WAXS data, which provides structural information on a scale of tens to several angstroms, reveals a shoulder peak centered at 0.35 Å^−1^, corresponding to the scattering interference between adjacent Zr‐clusters within the agglomerates. This indicates an inter‐Zr‐cluster distance of 17.9 Å. Another peak at 1.4 Å^−1^, corresponding to an average correlation distance of 4.5 Å, is assigned to scattering from the Zr‐cluster's local coordination structures, consistent with the XRD data.

Macroscopic weight‐loading tests highlighted the remarkable mechanical strength of poly(UIO‐TA). No deformation or fracture occurred when a 2.0 kg weight was suspended from a sample of poly(UIO‐TA) (Figure 2f). Nanoindentation load–displacement curves were obtained under a maximum indentation load of 1750 µN (Figure 2g). The calculated elastic modulus and hardness were 0.39 and 0.024 GPa, respectively, underscoring the robust binding strength of the zirconium(IV)‐carboxylate coordination bonds. The indentation curve for poly(UIO‐TA) displayed hysteresis, similar to that typically observed in ceramics or metals. However, the mechanical strength of poly(UIO‐TA) decreased significantly when the temperature exceeded 120 °C. Temperature‐dependent rheological measurements further revealed that the copolymer's viscosity decreased significantly from 8.0 × 10^5^ to 5.2 × 10⁴ Pa·s as the temperature increased from 25 to 120 °C (Figure 2h; Figure S11, Supporting Information). This significant viscosity‐temperature dependence allows for easy processing and molding of poly(UIO‐TA).

### 2.2 Adhesion Performance and Mechanism of Poly(UIO‐TA)

**Figure 3:**
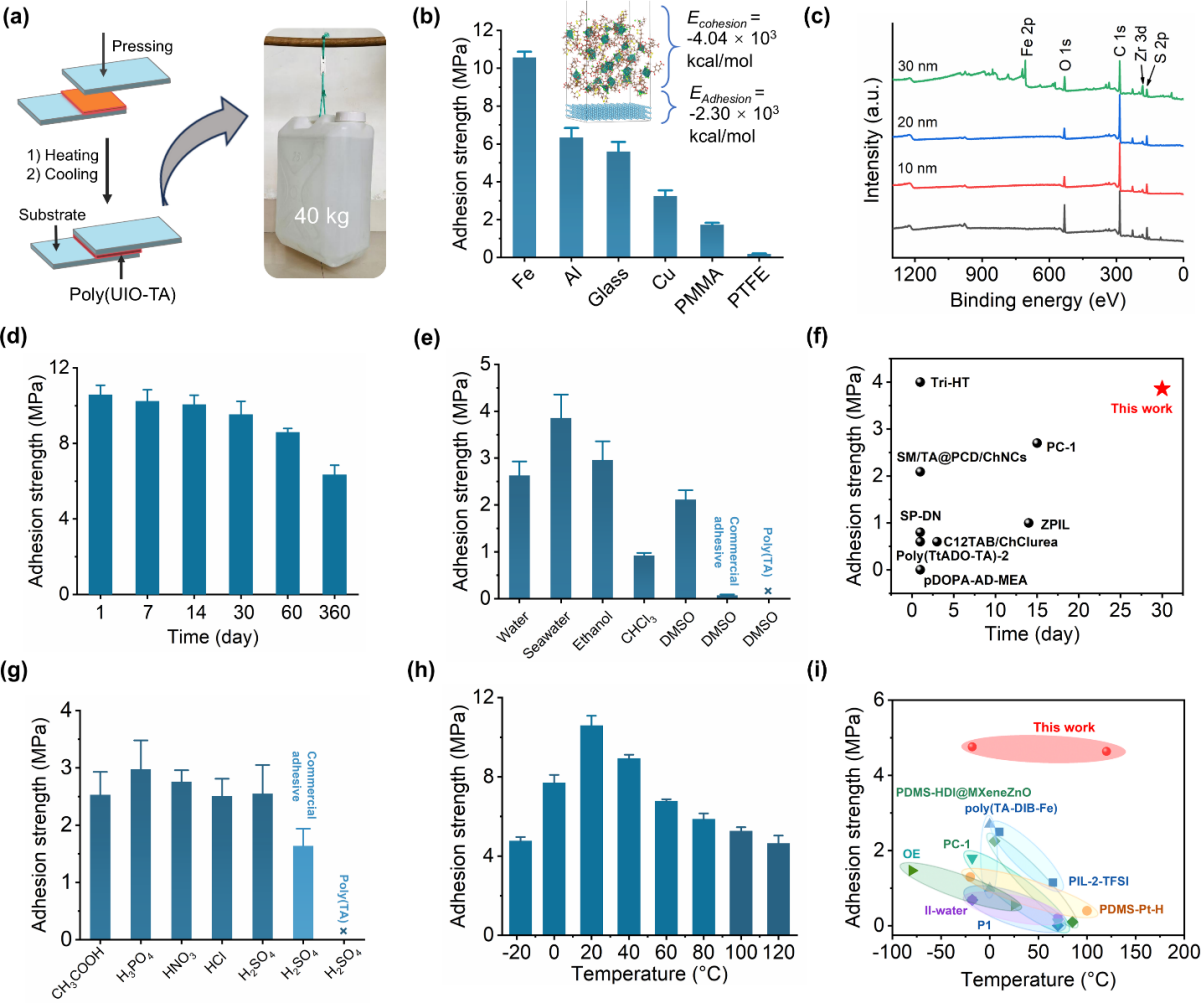
Adhesion performance of poly(UIO‐TA). a) Macroscopic adhesion tests of poly(UIO‐TA). b) Adhesion strengths of poly(UIO‐TA) on various substrates. c) XPS depth profiling of poly(UIO‐TA) adhesion layer on steel surface. d) Time‐dependent adhesion strength of poly(UIO‐TA) on steel. e) Adhesion strengths of poly(UIO‐TA), commercial adhesive, and poly(TA) on steel in various solutions over 4 weeks. f) Comparison of adhesion strength of poly(UIO‐TA) and reported adhesives after soaking in organic solvents for different times. g) Adhesion strengths of poly(UIO‐TA), commercial adhesive, and poly(TA) on steel in various acids over 4 weeks. h) Temperature‐dependent adhesion strength of poly(UIO‐TA) on steel. i) Comparison of temperature‐dependent adhesion strength of poly(UIO‐TA) with reported adhesives. Detailed data can be found in Tables S1 and S2 (Supporting Information). For panels (b), (d), (e), and (g), tests were conducted at 25 °C. Error bars represent the standard deviations of adhesion strengths of poly(UIO‐TA) (*n* = 3).

Thanks to the in situ spontaneous polymerization, the substrate surface (e.g., glass) underwent isothermal curing, followed by hot pressing with a second substrate at 130 °C. After cooling to ambient temperature, an adhesive interlayer, ≈15 µm thick, was formed, ensuring a strong and intimate bond between the two substrates (**Figure** **3**a). Without the need for organic solvents or complex resolidification processes, an ultrathin adhesive layer (15 µm thick, 8–10 mg·cm^−2^) of poly(UIO‐TA) was formed by simply depositing it onto one substrate and hot pressing with a second substrate. This thin poly(UIO‐TA) layer exhibited remarkable adhesion performance. For example, an adhesive area of 12 cm^2^ on steel, achieved through hot‐melt pretreatment, successfully supported a load of 40 kg without any detachment.

Lap shear tests were conducted to quantitatively evaluate the adhesion performance of poly(UIO‐TA) across various substrates. The material demonstrated excellent adhesion to a range of surfaces. As shown in Figure 3b, poly(UIO‐TA) achieved an impressive adhesion strength of 10.58 MPa on steel (Fe) at room temperature, surpassing both commercial adhesives and previously reported supramolecular adhesives.^41^ On hydrophobic substrates such as polymethylmethacrylate (PMMA), the adhesion strength was 1.73 MPa, while on polytetrafluoroethylene (PTFE), known for its ultralow surface energy, the adhesion strength was 0.19 MPa. In contrast, poly(TA) exhibited low adhesion strength of 1.41 MPa on steel surface (Figure S14, Supporting Information). UIO‐TA is a solid powder with no adhesive properties. In addition, the adhesion strength of hybrid materials can be modulated by adjusting the concentration of TA in the polymer network (Figures S15–S19, Supporting Information). Additionally, poly(UIO‐TA) exhibited outstanding long‐term adhesion performance. After 12 months, adhesion strengths on steel and glass were measured at 6.34 and 4.00 MPa, respectively, retaining 60%–70% of the initial adhesion strength (Figure 3d; Figure S20, Supporting Information). The adhesion strength remained strong at 3.56 MPa after UV irradiation for seven days (Figure S21, Supporting Information).

The high adhesion performance could be attributed to the synergistic effect of i) strong noncovalent interactions (e.g., metal coordination bonds, hydrogen bonds) at the interface, and ii) robust and durable SPNs (Figures S22 and S23, Supporting Information). To validate this hypothesis, X‐ray photoelectron spectroscopy (XPS) analysis of poly(UIO‐TA) on steel surface was performed. XPS depth profiling revealed no significant change in the intensity of the S 2p peak throughout the etching process from the bulk to the interface, confirming the uniform distribution of sulfur across both regions (Figure 3c). This also suggests that poly(UIO‐TA) exhibits high penetration capability, enabling molecule‐level interaction with surfaces and promoting the formation of strong interfacial bonds. Furthermore, the XPS results showed that the S 2p and Zr 3d peaks vanish after the adhesion layer on steel is immersed in sodium hydroxide solution (Figure S23, Supporting Information). This indicates that the strong interfacial adhesion is due to noncovalent interactions between the carbonyl groups and the metal atoms at the interface, rather than covalent bonding. Density functional theory (DFT) calculations confirmed strong cohesive energy (4.0 × 10^3 ^kcal mol^−1^) and interfacial adhesion energy between poly(UIO‐TA) and steel surface (2.3 × 10^3 ^kcal mol^−1^) (Figure 3b).

### 2.3 Adhesion Behavior of Poly(UIO‐TA) in Harsh Environment

Poly(UIO‐TA) exhibits excellent stability in a variety of solvents, including water, simulated seawater, and common organic solvents such as ethanol, n‐hexane, dimethyl sulfoxide (DMSO), trichloromethane (CHCl_3_), toluene, and acetone. No significant swelling was observed after four weeks of immersion in these solvents (Figure S24, Supporting Information). In contrast, poly(TA) dissolves in ethanol and DMSO after soaking for 2 h. To further explore the solvent‐resistant adhesive behavior, poly(UIO‐TA) was immersed in deionized water and simulated seawater. As shown in Figure S25 (Supporting Information), the material exhibited robust adhesion to steel surface after immersion. For example, after 24 h in deionized water, the adhesion strength reached 5.06 MPa, retaining 85% of its original strength. Even after four weeks of immersion, the adhesion strength remained high at 2.63 MPa. Similarly, strong and lasting adhesion was observed after immersion in common organic solvents, including nonpolar (CHCl_3_), polar (ethanol), and highly solvating solvents (DMSO). Notably, poly(UIO‐TA) retained 2.11 MPa of adhesion strength after one month in DMSO, outperforming most supramolecular adhesives and commercial adhesives (Figure 3e).^[^ ^22^ ^]^ For comparison, commonly used commercial adhesives, such as epoxy resin and acrylate adhesives, degrade upon immersion in DMSO within 24 h, leading to a significant loss in adhesion strength or complete failure (Figure 3f).

It is impressive that poly(UIO‐TA) demonstrates robust and long‐term durability in both inorganic and organic acids, including hydrochloric acid (HCl), sulfuric acid (H_2_SO_4_), nitric acid (HNO_3_), phosphoric acid (H_3_PO_4_), and acetic acid (CH_3_COOH). After 24 h of immersion in an HCl solution (0.1 mol L^−1^), no adhesion failure was observed on the bonded steel substrate. As shown in Figure 3g, the adhesion strength of poly(UIO‐TA) remained stable at 2.51 and 2.76 MPa on steel after four weeks of exposure to HCl and HNO_3_ solution, respectively. Moreover, the adhesion strength of poly(UIO‐TA) on steel surface (2.55 MPa) in H_2_SO_4_ solution (0.1 mol L^−1^) exceeds that of commercial epoxy resin adhesives (1.64 MPa) and most reported supramolecular adhesives (<1.0 MPa). Notably, poly(UIO‐TA) demonstrates an adhesion strength of up to 2.53 MPa in acetic acid, maintaining 25% of its original adhesion strength compared to the untreated condition. In contrast, the reference poly(TA) was unsuitable for use under acidic conditions. This enhanced stability can be attributed to the metal coordination interactions that drive the stable self‐assembly of poly(UIO‐TA).

Temperature has a significant impact on the adhesion performance of poly(UIO‐TA). The temperature‐dependent adhesion behavior was systematically investigated, and the material demonstrated robust and durable adhesion across a temperature range of −20 to 120 °C (Figure S28, Supporting Information). For example, no noticeable detachment of the steel substrate (with an adhesion area of 0.7 cm^2^) occurred after 2 h at 100 °C, even under a 2 kg load. As shown in Figure 3h, the adhesion strength of poly(UIO‐TA) on steel was 5.86 MPa at 80 °C for 2 h. At 120 °C, the adhesion strength remained strong at 4.64 MPa, surpassing that of most previously reported supramolecular adhesives, which typically exhibit strengths of ≈2.70 MPa (Figure 3i).^[^ ^24^ ^]^ In contrast, when the temperature exceeded 100 °C, the adhesion strength of poly(TA) on the steel surface decreased significantly, whereas poly(UIO‐TA) maintained much higher adhesion strength. This suggests that the incorporation of a noncovalent network, formed through stepwise coordination‐driven assembly, enhances the high‐temperature stability of poly(UIO‐TA). Additionally, poly(UIO‐TA) demonstrated strong adhesion even at low temperatures. For example, at −18 °C, its adhesion strength on steel remained at 4.76 MPa. As shown in Figure S29 (Supporting Information), poly(UIO‐TA) demonstrated stable adhesion performance under varying relative humidity levels (25% RH or 50% RH), maintaining a strength of 10.59 MPa without significant changes. After prolonged exposure to 50% RH for 12 months, the adhesion strength slightly decreased to 6.34 MPa. In a higher‐humidity environment (e.g., 95% RH), the adhesion strength further reduced, reaching 5.80 MPa at 95% RH. Overall, poly(UIO‐TA) exhibited robust and durable adhesion strength in harsh environments, including seawater, organic solvents, acidic solutions, wide temperature range (i.e., −20 to 120 °C), and high relative humidity. This further expands the potential applications of supramolecular adhesives.

### 2.4 Improvement of Adhesion Strength Through Cyclic Adhesion

**Figure 4:**
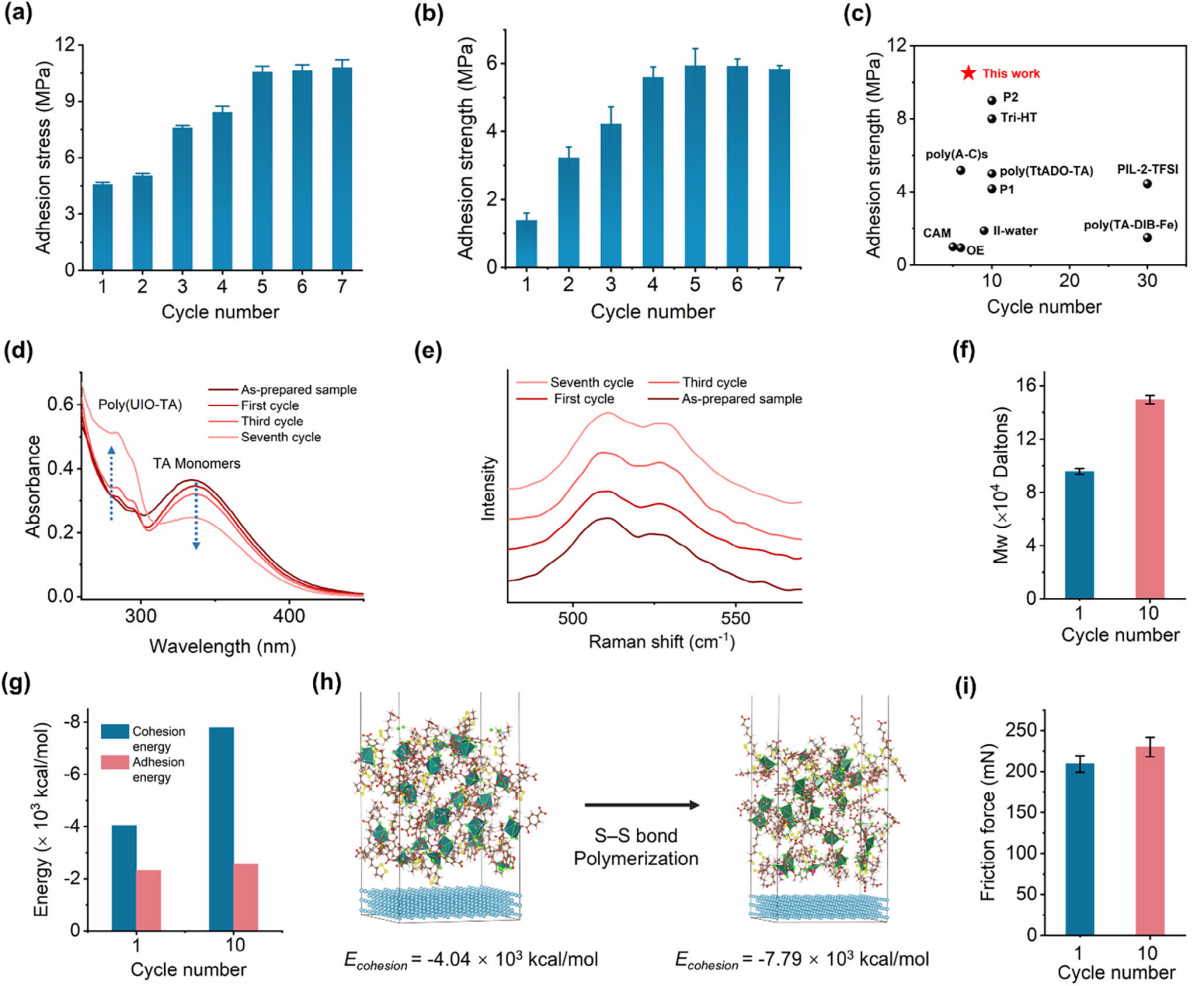
Cyclic adhesion performance of poly(UIO‐TA). a) Cyclic adhesion performance of poly(UIO‐TA) on steel at room temperature. b) Cyclic adhesion performance of poly(UIO‐TA) on glass at room temperature. c) Comparison of adhesion strength and cycle number for different adhesives (Table S3, Supporting Information). d) UV–vis spectra of poly(UIO‐TA) after cyclic adhesion testing. e) Raman spectra of poly(UIO‐TA) after cyclic adhesion testing. f) Molecular weight (Mw) of poly(UIO‐TA) before and after 10 cycles. g) Cohesion energy and adhesion energy of poly(UIO‐TA) before and after 10 cycles, calculated by MD simulation. h) Molecular dynamic simulations of poly(UIO‐TA) and steel (with Fe atom as the substrate model). i) Friction force of poly(UIO‐TA) on steel before and after 10 cycles, evaluated by nanoscratch test. Error bars represent the standard deviations (n = 3).

In general, for the majority of reversible adhesives, the adhesive strength remains constant or diminishes as the cycle number increases. However, the adhesion strength of poly(UIO‐TA) gradually increased and plateaued after multiple cycling experiments (**Figure** **4**a,b). Specifically, the adhesion strength on steel increased from 4.59 to 10.58 MPa during the first five cycles and then stabilized at ≈10.58 MPa. The maximum value of 10.58 MPa was higher than that of most previously reported supramolecular polymer adhesives (Figure 4c; Figure S30, Supporting Information). A similar trend was observed for the adhesion of poly(UIO‐TA) to glass, which can be primarily attributed to the in situ spontaneous polymerization of dynamic disulfide bonds. To verify this hypothesis, polymer formation was tracked by Ultraviolet–visible (UV–vis) spectroscopy of poly(UIO‐TA) during the cyclic lap‐shear process (Figure 4d). The absorption peak at ≈269 nm, corresponding to poly(TA), increased in intensity with the number of cycles, suggesting gradual disulfide ring‐opening polymerization.^[^ ^45^ ^]^ Additionally, Raman spectroscopy further confirmed the polymerization. As shown in Figure 4e, the 507 cm^−1^ peak for disulfide bonds split into two pronounced peaks, validating the ring‐opening polymerization of TA.^[^ ^45^ ^]^ The new peak at 525 cm^−1^ emerged and its intensity progressively increased with cycle numbers, indicating a rise in the ring‐opening conversion rate of TA. In addition, the weight‐average Mw of poly(UIO‐TA) after cyclic adhesion tests was determined by gel permeation chromatography. As shown in Figure 4f, the Mw increased from 9.6 × 10^4^ daltons to 1.5 × 10^5^ daltons after 10 cycles, indicating that the dynamic disulfide bonds within poly(UIO‐TA) underwent polymerization, leading to the formation of a denser SPN.

Additionally, the complex viscosity of poly(UIO‐TA) was measured using a rheometer. As shown in Figure 2h, an increase in complex viscosity was observed after the rheological cyclic tests, indicating the formation of a high‐density polymeric network. The adhesion strength is determined not only by the cohesive energy but also by the interfacial adhesion strength. Thus, the cohesion energy and adhesion energy of UIO‐TA and poly(UIO‐TA) were calculated using molecular dynamics simulations. The results revealed that poly(UIO‐TA) exhibited a higher cohesion energy (−7.79 kcal·mol^−1^) compared to UIO‐TA (−4.04 kcal·mol^−1^) (Figure 4g,h). In contrast, only a slight increase in the adhesion energy between poly(UIO‐TA) and the steel surface was observed relative to UIO‐TA. Furthermore, the adhesion force between poly(UIO‐TA) and the steel surface was evaluated through nanoscratch testing. After 10 cycles of adhesion tests, the adhesion force of poly(UIO‐TA) showed no significant increase (Figure 4i). Therefore, the enhanced adhesion strength of poly(UIO‐TA) on substrate surfaces (e.g., steel and glass) can primarily be attributed to the increased cohesion energy resulting from the further open‐ring polymerization of dynamic disulfide bonds.

## 3 Conclusion

In conclusion, we have developed a simple, room‐temperature synthesis method for metal‐coordinated nanoparticles, which can self‐assemble into SPNs with an interconnected porous structure. These networks are formed via both dynamic covalent and noncovalent crosslinking. Poly(UIO‐TA) successfully integrates the exceptional reversibility of the noncovalent network with the high strength of the dynamic covalent network, resulting in a material that combines high mechanical strength with excellent reprocessability. Poly(UIO‐TA) demonstrates outstanding adhesion performance, exhibiting strong, tough, and durable adhesion under mild conditions, with an adhesion strength reaching up to 10.59 MPa. Moreover, it maintains robust adhesion even in harsh environments, including elevated temperatures (120 °C), water, simulated seawater, both nonpolar and polar organic solvents, and strong acids such as hydrochloric and nitric acid. This exceptional performance is attributed to the unique hybrid structure of the SPNs, which provides both thermal stability and excellent solvent resistance. Furthermore, the adhesion strength of poly(UIO‐TA) increased by ≈2.5 times after five cycles of adhesion testing, primarily due to the enhanced cohesion energy resulting from the further polymerization of dynamic disulfide bonds. Overall, this work paves the way for the development of supramolecular polymer materials with outstanding stability and superior adhesion performance.

## Conflict of Interest

The authors declare no conflict of interest.

## Supporting information



Supporting Information

## Data Availability

The data that support the findings of this study are available in the supplementary material of this article.

## References

[1] G. P. Maier , M. V. Rapp , J. H. Waite , J. N. Israelachvili , A. Butler , Science 2015, 349, 628. 26250681 10.1126/science.aab0556

[2] J. Saiz‐Poseu , J. Mancebo‐Aracil , F. Nador , F. Busqué , D. Ruiz‐Molina , Angew. Chem., Int. Ed. 2019, 58, 696. 10.1002/anie.201801063 29573319

[3] S. Wu , C. Cai , F. Li , Z. Tan , S. Dong , Angew. Chem., Int. Ed. 2020, 59, 11871. 10.1002/anie.202004104 32291882

[4] J. Park , J. Park , J. Lee , C. Lim , D. W. Lee , Nat. Commun. 2022, 13, 112. 35013244 10.1038/s41467-021-27659-w PMC8748952

[5] C. Shi , Q. Zhang , B. Wang , D. He , H. Tian , D.‐H. Qu , CCS Chem. 2023, 5, 1422.

[6] J. Liu , Y.‐S. Huang , Y. Liu , D. Zhang , K. Koynov , H.‐J. Butt , S. Wu , Nat. Chem. 2024, 16, 1024. 38459235 10.1038/s41557-024-01476-2 PMC11164683

[7] H. Ju , Z. Yin , Z. Demchuk , V. Bocharova , C. Gainaru , J. A. Laub , K. Vogiatzis , R. Advincula , J. Chen , P.‐F. Cao , Adv. Funct. Mater. 2024, 34, 2402165.

[8] T. Nogusa , C. B. Cooper , Z. Yu , Y. Zheng , Y. Shi , Z. Bao , Matter 2023, 6, 2439.

[9] Y. Li , P. Sun , J.‐F. Xu , X. Zhang , ACS Mater. Lett. 2023, 5, 2528.

[10] Y. Wang , G. Liu , J. Zhao , Z. Zhang , H. Zhang , Y. Ding , X. Zhang , Z. Liu , W. Yu , X. Yan , Angew. Chem., Int. Ed. 2024, 63, 202409705. 10.1002/anie.202409705 39072904

[11] G. Finkelstein‐Zuta , Z. A. Arnon , T. Vijayakanth , O. Messer , O. S. Lusky , A. Wagner , G. Zilberman , R. Aizen , L. Michaeli , S. Rencus‐Lazar , S. Gilead , S. Shankar , M. J. Pavan , D. A. Goldstein , S. Kutchinsky , T. Ellenbogen , B. A. Palmer , A. Goldbourt , M. Sokol , E. Gazit , Nature 2024, 630, 368. 38867128 10.1038/s41586-024-07408-x

[12] J. Feng , Z. Lin , Y. Zhang , L. Fang , Q. Zhu , D. Yu , Angew. Chem., Int. Ed. 2024, 63, 202411815. 10.1002/anie.202411815 39032126

[13] Q. Deng , S. Han , Y. Wu , Y. Chen , Y. Zhang , Y. Zhao , S. Chen , J. Zhu , Angew. Chem., Int. Ed. 2024, 64, 202415386. 10.1002/anie.202415386 39450609

[14] Y. Zhao , S. Song , X. Ren , J. Zhang , Q. Lin , Y. Zhao , Chem. Rev. 2022, 122, 5604. 35023737 10.1021/acs.chemrev.1c00815

[15] J. Zhang , W. Wang , Y. Zhang , Q. Wei , F. Han , S. Dong , D. Liu , S. Zhang , Nat. Commun. 2022, 13, 5214. 36064871 10.1038/s41467-022-32997-4 PMC9445047

[16] D. Gao , G. Thangavel , J. Lee , J. Lv , Y. Li , J.‐H. Ciou , J. Xiong , T. Park , P. S. Lee , Nat. Commun. 2023, 14, 1990. 37031201 10.1038/s41467-023-37535-4 PMC10082814

[17] Y. Liu , L. Wang , L. Zhao , Y. Zhang , Z.‐T. Li , F. Huang , Chem. Soc. Rev. 2024, 53, 1592. 38167687 10.1039/d3cs00705g

[18] S. Wu , C. Cai , F. Li , Z. Tan , S. Dong , CCS Chem. 2021, 3, 1690.

[19] S. Wu , W. Wang , C. Cai , F. Li , S. Dong , Chin. Chem. Lett. 2023, 34, 107830.

[20] W. Guan , W. Jiang , X. Deng , W. Tao , J. Tang , Y. Li , J. Peng , C.‐L. Chen , K. Liu , Y. Fang , Angew. Chem., Int. Ed. 2023, 62, 202303506. 10.1002/anie.202303506 37016787

[21] C. Heinzmann , C. Weder , L. M. de Espinosa , Chem. Soc. Rev. 2016, 45, 342. 26203784 10.1039/c5cs00477b

[22] C. Cui , W. Liu , Prog. Polym. Sci. 2021, 116, 101388.

[23] S. Chen , Z. Li , Y. Wu , N. Mahmood , F. Lortie , J. Bernard , W. H. Binder , J. Zhu , Angew. Chem., Int. Ed. 2022, 61, 202203876. 10.1002/anie.202203876 35426214

[24] C. Cai , H. Gong , S. Wu , F. Li , S. Liu , Z. Tan , S. Dong , Chem. Eng. J. 2023, 451, 138674.

[25] J. Zhang , X. Zhou , Q. Hu , K. Zhou , Y. Zhang , S. Dong , G. Zhao , S. Zhang , Nat. Commun. 2024, 15, 4265. 38769305 10.1038/s41467-024-48561-1 PMC11106314

[26] J. Xiong , M. Duan , X. Zou , S. Gao , J. Guo , X. Wang , Q. Li , W. Li , X. Wang , F. Yan , J. Am. Chem. Soc. 2024, 146, 13903. 38721817 10.1021/jacs.4c01758

[27] L. He , Y. Jiang , J. Wei , Z. Zhang , T. Hong , Z. Ren , J. Huang , F. Huang , P. J. Stang , S. Li , Nat. Commun. 2024, 15, 3050. 38594237 10.1038/s41467-024-47333-1 PMC11004166

[28] A. L. Dobson , N. J. Bongiardina , C. N. Bowman , ACS Appl. Polym. Mater. 2020, 2, 1053. 34079938 10.1021/acsapm.9b00992 PMC8168480

[29] M. Wu , Y. Liu , P. Du , X. Wang , B. Yang , Int. J. Adhes. 2020, 100, 102597.

[30] Z. Liu , Y. Tang , Y. Chen , Z. Lu , Z. Rui , Chem. Eng. J. 2024, 497, 154710.

[31] S. Luo , N. Wang , Y. Pan , B. Zheng , F. Li , S. Dong , Small 2024, 20, 2310839. 10.1002/smll.202310839 38225689

[32] C. Wang , S. Huo , G. Ye , Q. Zhang , C. Cao , M. Lynch , H. Wang , P. Song , Z. Liu , Chem. Eng. J. 2024, 500, 157418.

[33] K. Zhou , Q. Zhang , J. Gong , H. Shen , H. Luo , S. Chen , X. Zhang , N. Zhang , X. Pei , T. Wang , Y. Yang , Q. Wang , Y. Zhang , ACS Appl. Mater. Interfaces 2024, 16, 64031. 39505404 10.1021/acsami.4c14965

[34] J. Zhao , Z. Zhang , C. Wang , X. Yan , CCS Chem. 2024, 6, 41.

[35] L. Liu , Y. Deng , D.‐H. Qu , B. L. Feringa , H. Tian , Q. Zhang , Angew. Chem., Int. Ed. 2025, 64, 202424147. 10.1002/anie.202424147 39808487

[36] M. A. Rahman , C. Bowland , S. Ge , S. R. Acharya , S. Kim , V. R. Cooper , X. C. Chen , S. Irle , A. P. Sokolov , A. Savara , T. Saito , Sci. Adv. 2021, 7, abk2451. 10.1126/sciadv.abk2451 PMC8519568 34652933

[37] Z. Zhao , P. Zhao , Y. Zhao , J. Zuo , C. Li , Adv. Funct. Mater. 2022, 32, 2201959.

[38] S. Chen , J. T. Aladejana , X. Li , M. Bai , S. Q. Shi , H. Kang , J. Cao , J. Li , A. Strong , Ind. Crops. Prod. 2023, 194, 116277.

[39] C. Shi , Q. Zhang , H. Tian , D.‐H. Qu , SmartMat. 2020, 1, 1012.

[40] Q. Zhang , Y. Deng , H. Luo , C.‐Y. Shi , G. M. Geise , B. L. Feringa , H. Tian , D.‐H. Qu , J. Am. Chem. Soc. 2019, 141, 12804. 31348651 10.1021/jacs.9b05740 PMC6696886

[41] W. Zhao , J. Tropp , B. Qiao , M. Pink , J. D. Azoulay , A. H. Flood , J. Am. Chem. Soc. 2020, 142, 2579. 31931561 10.1021/jacs.9b12645

[42] W. Zhang , N. Pinna , Chem. –Eur. J. 2024, 30, 202304256. 10.1002/chem.202304256 38300687

[43] H. Li , H. Shi , X. Chen , Z. Ren , Y. Shen , P. Wu , Y. Fan , X. Zhang , W. Shi , H. Liao , S. Zhang , W. Zhang , F. Huo , Adv. Mater. 2023, 35, 2209777. 10.1002/adma.202209777 36493462

[44] Z. Mu , K. Kong , K. Jiang , H. Dong , X. Xu , Z. Liu , R. Tang , Science 2021, 372, 1466.

[45] Q. Zhang , Y. Deng , C.‐Y. Shi , B. L. Feringa , H. Tian , D.‐H. Qu , Matter 2021, 4, 1352.

