# Supplementary material for: Robust and Reversible Supramolecular Adhesive via Dynamic Covalent Bond Crosslinking‐Induced Assembly of Metal‐Coordinated Nanoparticles
Source: Adv Sci (Weinh). 2025 Jul 30;12(39):e05122. doi: 10.1002/advs.202505122 (PMC12533303; doi:10.1002/advs.202505122)
Supplement: Supplementary file 1 — Supporting Information [file ADVS-12-e05122-s001.docx]

Supporting Information

For

**Robust and Reversible Supramolecular Adhesive via Dynamic Covalent Bond Crosslinking-Induced Assembly of Metal-Coordinated Nanoparticles**

Yanyan Guo,^[a]^ Mengran Zhang,^[a]^ Shuanggen Wu,*^,[a]^ and Xunqiu Wang*^,[a]^

^[a]^ State Key Laboratory of Coking Coal Resources Green Exploitation, School of Chemical Engineering, Zhengzhou University, Zhengzhou 450001, P. R. China
*Correspondence: Shuanggen Wu, wu[shuanggen@zzu.edu.cn](mailto:shuanggenwu@zzu.edu.cn); Xunqiu Wang, wxqiu@zzu.edu.cn

**Table of Contents**

[1. Materials and methods S1](#_Toc203401225)

[2. Preparation of UIO-TA and poly(UIO-TA) S2](#_Toc203401226)

[3. Adhesion experiment and measurement S2](#_Toc203401227)

[4. DFT calculation and molecular dynamics simulation S2](#_Toc203401228)

[5. Time-dependent UV-vis spectra of UIO-TA S4](#_Toc203401229)

[6. XPS spectra Zr 3d in UIO and UIO-TA. S5](#_Toc203401230)

[7. Positron annihilation lifetime spectroscopy (PALS) spectra of UIO and UIO-TA S6](#_Toc203401231)

[8. Time-dependent Raman spectra of poly(UIO-TA) S7](#_Toc203401232)

[9. Time-of-flight secondary ion mass spectrometry (TOF-SIMS) of poly(UIO-TA) S8](#_Toc203401233)

[10. Scanning electron microscopy (SEM) images of poly(UIO-TA) S10](#_Toc203401234)

[11. Rheology measurements of poly(UIO-TA) S11](#_Toc203401235)

[12. Thermal properties of poly(UIO-TA) S12](#_Toc203401236)

[13. Long-term adhesion strength of poly(UIO-TA) S18](#_Toc203401237)

[14. Contact angle of poly(UIO-TA) S19](#_Toc203401238)

[15. X-ray photoelectron spectroscopy (XPS) spetra of poly(UIO-TA) S20](#_Toc203401239)

[16. Solubility tests of poly(UIO-TA) S21](#_Toc203401240)

[17. References S27](#_Toc203401241)

1. **Materials and methods**

All materials and reagents were used as received, without further purification. Zirconium (IV) chloride was purchased from Shanghai Macklin Biochemical Technology Co., Ltd. Terephthalic acid (BDC) was obtained from Shanghai Aladdin Biochemical Technology Co., Ltd. Thioctic acid (TA) was supplied by Adamas-Beta.

Fourier-transform IR (FT-IR) spectra were recorded using a VERTEX 70 infrared spectrometer, with 32 scans taken from 4000 to 400 cm^-1^ at a resolution of 4 cm^-1^. Backgrounds were determined using blank KBr plates. Raman spectra obtained with a micro-Raman spectrometer (Renishaw, inVia Reflex) with a 785 nm excitation wavelength. Powder X-ray diffraction (PXRD) patterns were collected on a Rigaku SmartLab SE with Cu Kα radiation operating at 40 kV, over a 2θ range of 5–90^o^ at a scanning speed of 10^o^ min^-1^. Scanning electron microscopy (SEM) images were collected on ZEISS Sigma 300. Transmission electron microscope (TEM) images obtained with a JEM-1200EX microscope operating at 100 kV. Time-of-flight secondary ion mass spectrometry (TOF-SIMS) positive ion mass spectra and images were recorded using a PHI nanoTOFII TOF-SIMS, equipped with a 30 keV Bi^3+^ primary ion source. The pulsed target current was set at 2 nA. TOF-SIMS data were obtained and analyzed using the SurfaceLab 6.7 software. All positive mass spectra were calibrated to C^+^, C_2_H_3_^+^ and C_3_H_3_^+^.

Thermogravimetric (TG) analysis was performed using a NETZSCH STA 449 F3 apparatus, with a heating rate of 10°C min^-1^ over a temperature range of 30 to 800°C in a nitrogen atmosphere. Differential scanning calorimetry (DSC) measurements were performed using a TA Q200 at a heating rate of 10 °C min^-1^ within a temperature range of 30 to 200°C in a nitrogen environment. Small-angle X-ray scattering (SAXS) analyses were carried out using a D8 Discover X-ray Scattering system. Rheological assessments were performed with an Anton Paar MCR 92 rheometer, utilizing a PP15 model with a 15 mm diameter and a 1 mm gap setting. Argon adsorption isotherms was obtained using a Micromeritics ASAP 2460 instrument at 298.15 K. The specific surface area and pore size distribution of the samples were determined using the Brunauer-Emmett-Teller (BET) equation and the Density Functional Theory (DFT) method, respectively. X-ray photoelectron spectroscopy (XPS) experiments were conducted using a Thermo Scientific K-Alpha instrument. UV-Vis absorption spectra were collected using a Shimadzu UV-2700 spectrometer.

Nano-scratch testing was performed using a Hysitron TI-950 Nano Indenter. The number-average molecular weight (Mw) was determined via gel permeation chromatography (GPC) on a Shimadzu instrument, utilizing DMF as the eluent at a flow rate of 1 mL min^-1^, with an IR-20 refractive index detector for measurement. Ar sorption-desorption isotherms were measured at 87 K using a Micromeritics Tristar 3000 instrument. Contact angle measurements were executed with a contact angle goniometer (JCY-2). Positron annihilation lifetime spectroscopy (PALS) experiments were performed using a fast-fast coincidence system, with a 22Na source serving as the positron source, exhibiting an activity of approximately 10 mCi.^[1]^

1. **Preparation of UIO-TA and poly(UIO-TA)**

UIO-TA and poly(UIO-TA) were synthesized through a systematic procedure. Initially, ZrCl_4_ (0.0357 g) was dissolved in 10 mL of ethanol. Triethylamine (TEA) (0.4 mL) and 1,4-benzenedicarboxylic acid (BDC) (0.0164 g) were then added to the ethanol solution under vigorous stirring conditions. After 30 minutes, the molecular cluster UIO was obtained. A 10 mL ethanol solution of thioctic acid (TA) (7.6 mmol) was added dropwise to the UIO under vigorous stirring conditions. The UIO-TA was obtained after an additional 8 hours of continuous stirring. The resulting UIO-TA was then concentrated via centrifugation (H1650 Centrifuge, Cence) and washed three times with ethanol to remove any unreacted TA. Finally, the UIO-TA was dried in a vacuum oven at room temperature for over three days to yield poly(UIO-TA).

1. **Adhesion experiment and measurement**

Using the coating on the glass as a representative example, poly(UIO-TA) was coated on one glass slice, which was subsequently covered by another glass slice. The adhered glass pair was heated at 100°C for 30 minutes and then stored under various conditions before the pull-off adhesion tests. The amount of the adhesive layer was kept at approximately 10 mg cm^-2^. The accelerated weathering tests were conducted using a dark box UV analyzer, with a wavelength of 365 nm and a light source power of 90 W. Lap-shear tests were conducted using a universal testing machine (WDW-20, Jinan Star Test Technology) at a rate of 10 mm min^-1^. Each test was repeated three times to determine the standard deviation, which is represented by error bars.

1. **DFT calculation and molecular dynamics simulation**

The intermolecular interactions between Zr^4+^ and ligands such as TEA, BDC, and TA were calculated by density functional theory (DFT). The DFT calculation was carried out in the DMol3 package of Materials Studio 2018. The exchange-correlation potential was treated by using a mixed-generalized gradient approximation (m-GGA) with the Perdew-Burke-Ernzerhof (PBE) parametrization.^[2,3]^ A cutoff energy of 500 eV was employed, and the electronic energy was considered self-consistent when the energy change was smaller than 10^−5^ eV. The tolerance convergence in ionic was set to 10^-6^ eV. Additionally, the van der Waals correction of Grimme’s DFT-D3 model was adopted.^[4-6]^ The binding energy is calculated by the following formula:

*E*_be_ = *E*_total_ - (*E*_Zr_^4+^ + *E*_a_ + *E*_b_ + *E*_c_)

*E*_be_: Binding energy between Zr^4+^ and other groups.

*E*_total_: The total energy of systems.

*E*_Zr_^4+^: The energy of Zr^4+^.

*E*_a_~*E*_c_: Energy of different groups.

Cohesion energy and adhesion energy of UIO-TA and poly(UIO-TA) was calculated by molecular dynamics (MD) simulation. MD simulation was carried out in the Forcite package. In order to ensure the consistency with the experiment, a Harmonic functional form with 500 Kcal/mol/Å^2^ is added between some groups and the bonds. After the morphology and structure optimization of the obtained liquid phase model, five times of repeated annealing from 300 K to 500 K were performed. A relatively loose model is then obtained by relaxation of 100 ps through the NPT ensemble. The adsorption surface is constructed by cutting the Fe metal model along the (100) crystal surface. After the solid-liquid interface model is constructed, the final adsorption model is obtained through 1ns NVT relaxation. The interfacial adsorption energy is calculated by the following formula:

*E*_interfacial_ = *E*_total_ - (*E*_adhesive_ + *E*_Fe(100)_)

*E*_interfacial_: interaction energy between adhesive and Fe(100).

*E*_total_: The total potential energy of systems.

*E*_adhesive_: The potential energy of adhesive.

*E*_Fe(100)_: The potential energy of Fe(100).

1. **Time-dependent UV-vis spectra of UIO-TA**





**Figure S1.** Time-dependent UV-vis spectra of UIO-TA over a range of 0 to 8 hours.

Time-dependent UV-vis spectra of UIO-TA revealed that the concentration of TA initially decreased and then remained stable as time progressed, further indicating the successful grafting of TA onto the UIO-TA (Figure S1).

1. **XPS spectra Zr 3d in UIO and UIO-TA.**





**Figure S2.** XPS spectra of Zr 3d in UIO and UIO-TA.

XPS confirmed the shift of the Zr 3d peak from 182.4 eV in UIO to 182.6 eV in UIO-TA. The results indicated occupation of unsaturated Zr sites of UIO by the carboxyl group of TA.

1. **Positron annihilation lifetime spectroscopy (PALS) spectra of UIO and UIO-TA**


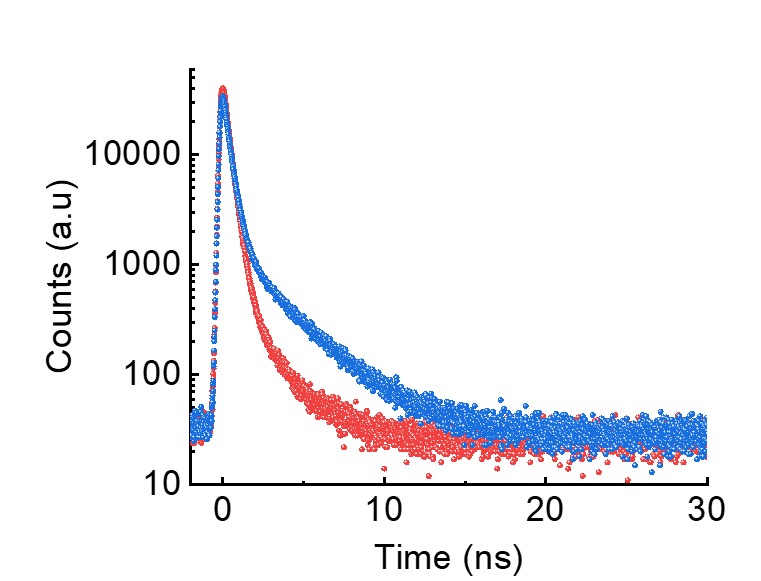


**Figure S3.** PALS spectra of UIO (red) and UIO-TA (blue).


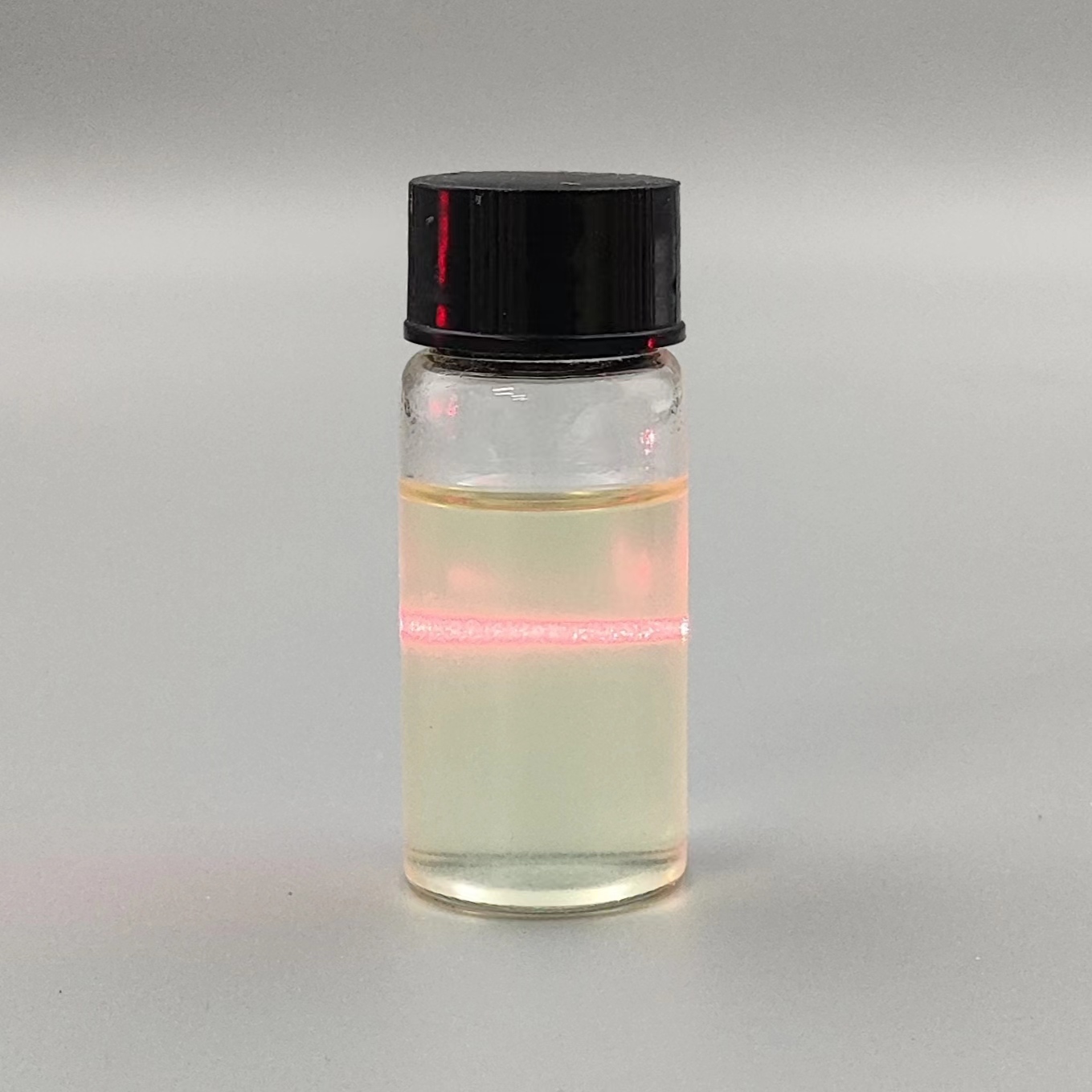


**Figure S4.** Tyndall light scattering of UIO-TA suspensions.

The Tyndall effect was observed during the preparation of UIO-TA, indicating the formation of UIO-TA nanoparticles (Figure S4).

1. **Time-dependent Raman spectra of poly(UIO-TA)**





**Figure S5**. Time-dependent Raman spectra of poly(UIO-TA) during the drying process.

1. **Time-of-flight secondary ion mass spectrometry (TOF-SIMS) of poly(UIO-TA)**


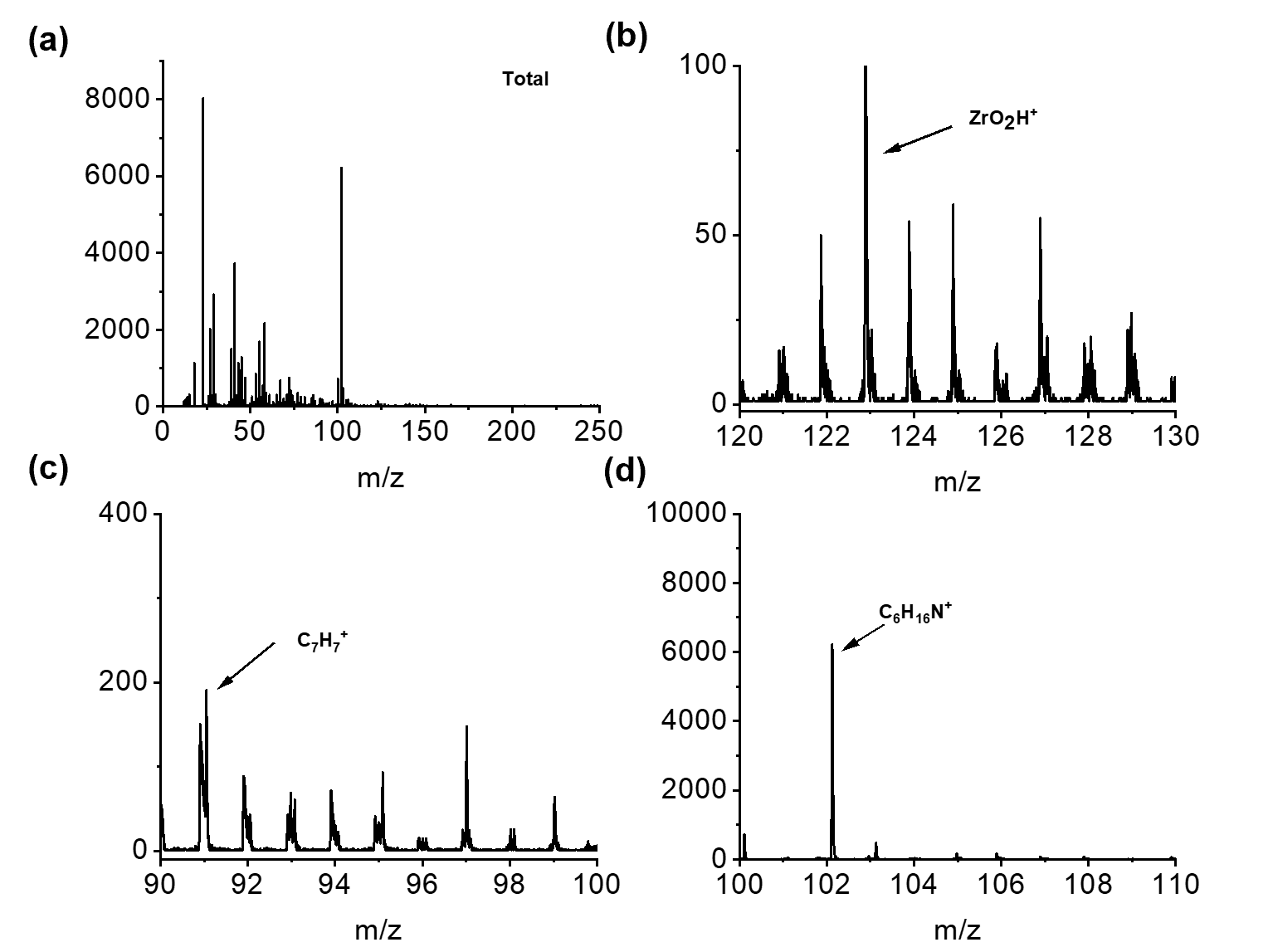


**Figure S6**. TOF-SIMS of the cation fragments of poly(UIO-TA). The intensity could not represent relative amounts of different molecular ion fragments due to their different ionization degrees.


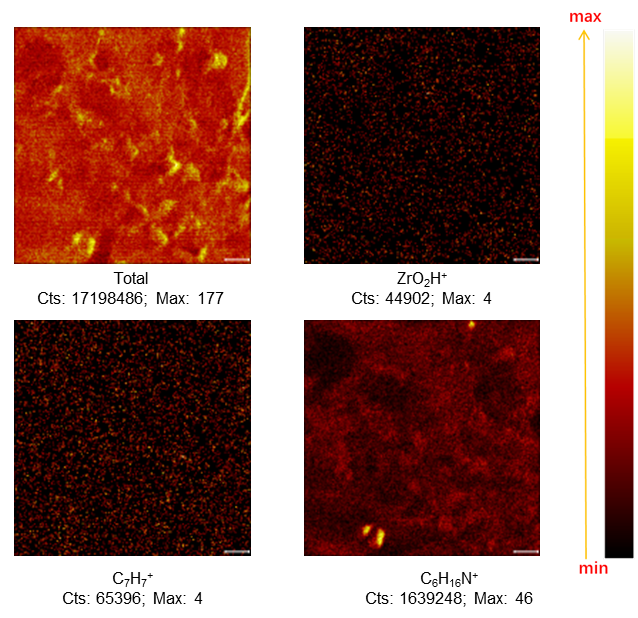


**Figure S7.** TOF-SIMS mapping images of the cation fragments of poly(UIO-TA). The intensity could not represent relative amounts of different molecular ion fragments due to their different ionization degrees.


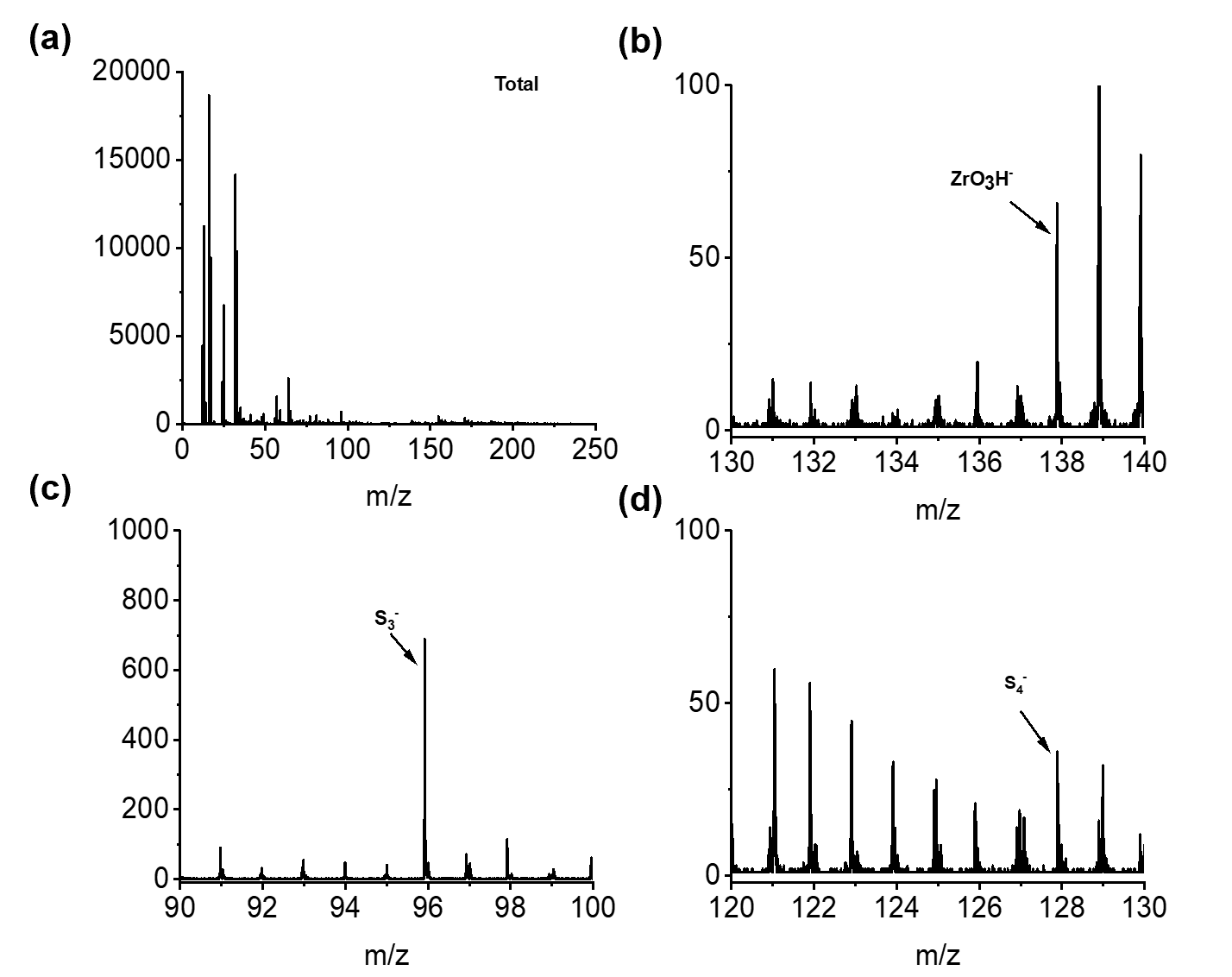


**Figure S8.** TOF-SIMS of the anion fragments of poly(UIO-TA). The intensity could not represent relative amounts of different molecular ion fragments due to their different ionization degrees.


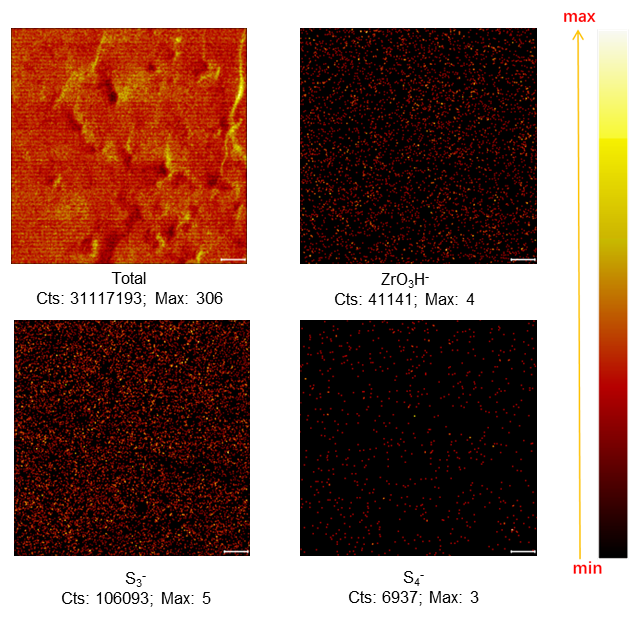


**Figure S9.** TOF-SIMS mapping images of the anion fragments of poly(UIO-TA). The intensity could not represent relative amounts of different molecular ion fragments due to their different ionization degrees.

1. **Scanning electron microscopy (SEM) images of poly(UIO-TA)**


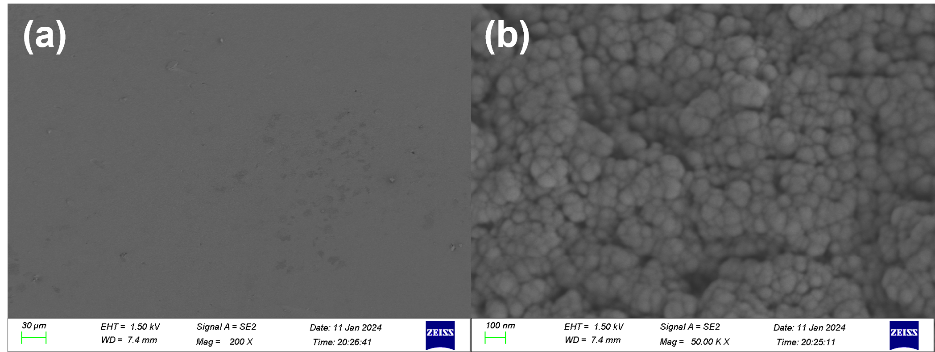


**Figure S10.** SEM images of poly(UIO-TA).

1. **Rheology measurements of poly(UIO-TA)**


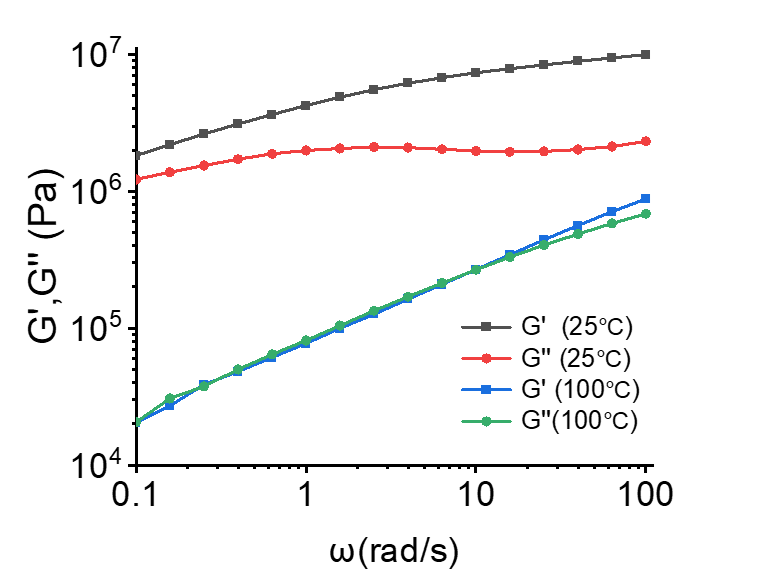


**Figure S11.** Storage modulus (*G’*) and loss modulus (*G’’*) of poly(UIO-TA) on frequency sweep at different temperatures.

Rheological experiments were conducted to explore the bulk properties and dynamically reconfigurable network of poly(UIO-TA) (Figure S11). In the rheological analysis, the storage modulus (*G*′) remained consistently higher than the loss modulus (*G*′′) across the entire frequency range, indicating the inherent stability and pronounced viscoelastic behavior of poly(UIO-TA). Temperature-dependent rheological measurements further demonstrated the effect of thermal exposure on the processing behavior of poly(UIO-TA) (Figure 2h). At elevated temperatures, the linear regions of both *G*′ and *G*′′ progressively narrowed, which can be attributed to the interplay of dynamic noncovalent interactions (e.g., hydrogen bonds and coordination bonds) and the restructuring of the disulfide bond backbone. Furthermore, the increase in temperature led to a decrease in the copolymer’s viscosity (5.2 × 10^4^ Pa·s at 120°C), facilitating the transition of poly(UIO-TA) from a solid to a liquid phase. This significant viscosity-temperature dependence allows for easy processing and molding of poly(UIO-TA).

1. **Thermal properties of poly(UIO-TA)**

**Figure S12.** TGA curves of poly(UIO-TA).


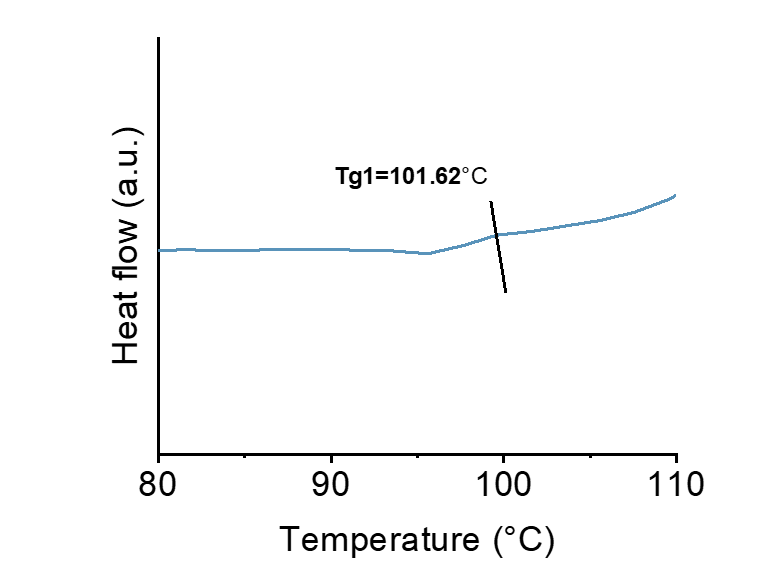


**Figure S13.** DSC curves of poly(UIO-TA).

The thermal properties of poly(UIO-TA) were systematically evaluated using thermal gravimetric analysis (TGA) and differential scanning calorimetry (DSC). The TGA results showed that the decomposition temperature (defined by a 5% weight loss) of poly(UIO-TA) exceeds 142°C, demonstrating its superior thermal stability (Figure S12). As shown in Figure S13, the DSC curve revealed that poly(UIO-TA) has a higher glass transition temperature (*T*_g_ = 101.6°C) compared to poly(TA) (*T*_g_ = -11°C), indicating that crosslinking of the UIO nanoparticles effectively restricts the mobility of the poly(TA) segment.





**Figure S14.** Adhesion strength of poly(TA) on various substrates at room temperature.

**Figure S15.** FI-TR spectra of poly(UIO-TA) obtained using different molar ratios of Zr^4+^ to TA. (a) n(Zr^4+^:TA) = 10:1; (b) n(Zr^4+^:TA) = 1:1; (c) n(Zr^4+^:TA) = 1:10; (d) n(Zr^4+^:TA) = 1:50; (e) n(Zr^4+^:TA) = 1:100.


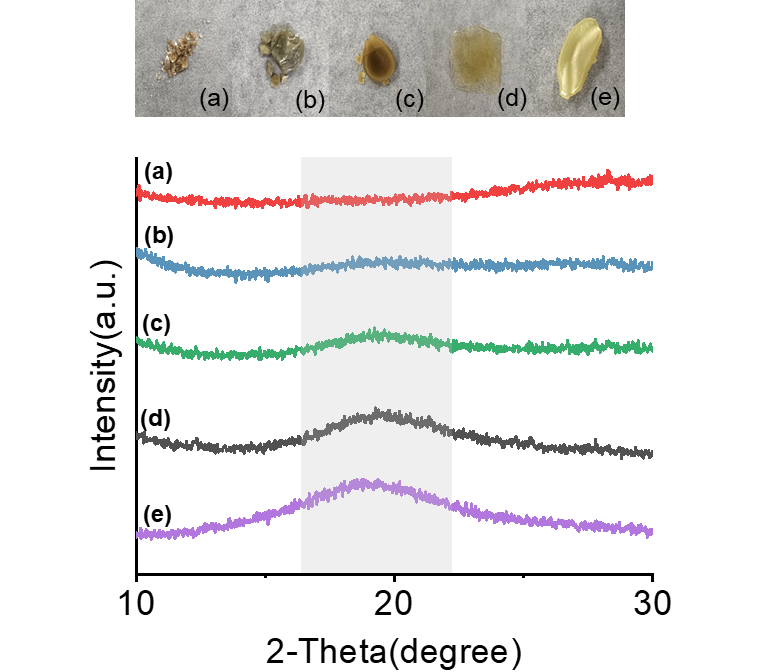


**Figure S16.** PXRD spectra of poly(UIO-TA) obtained using different molar ratios of Zr^4+^ to TA. (a) n(Zr^4+^:TA) = 10:1; (b) n(Zr^4+^:TA) = 1:1; (c) n(Zr^4+^:TA) = 1:10; (d) n(Zr^4+^:TA) = 1:50; (e) n(Zr^4+^:TA) = 1:100. The inset images show the macroscopic states of poly(UIO-TA). Notably, no crystalline peaks associated with TA were observed in any of the supramolecular polymer samples, suggesting the complete polymerization of TA. Furthermore, poly(UIO-TA) with varying concentrations of TA exhibited a consistently continuous, homogeneous, and amorphous structure.





**Figure S17.** TGA curves of poly(UIO-TA) obtained using different molar ratios of Zr^4+^ to TA.


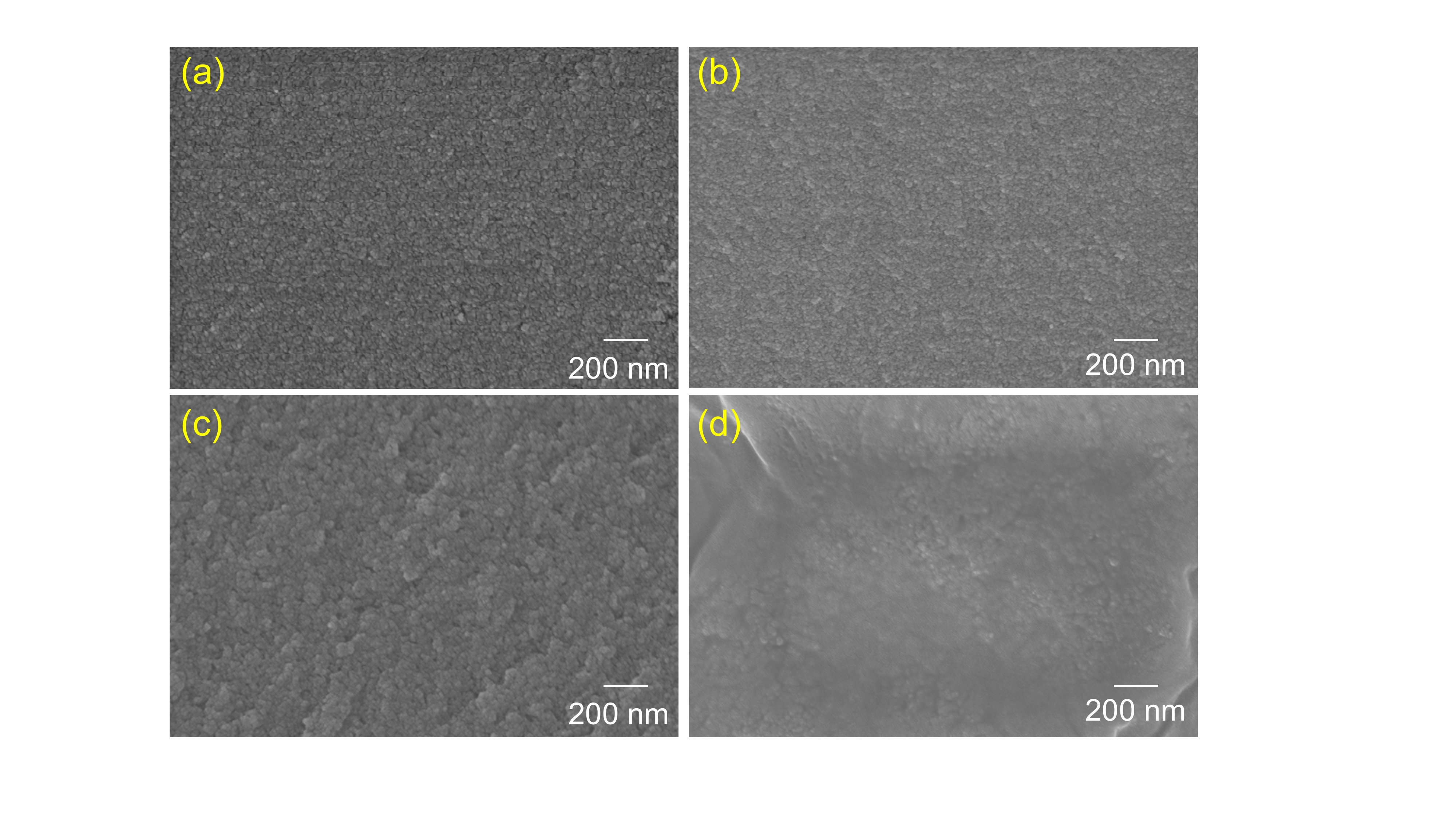


**Figure S18.** SEM images of poly(UIO-TA) obtained using different molar ratios of Zr^4+^ to TA. (a) n(Zr^4+^:TA) = 10:1; (b) n(Zr^4+^:TA) = 1:1; (c) n(Zr^4+^:TA) = 1:10; (d) n(Zr^4+^:TA) = 1:100.


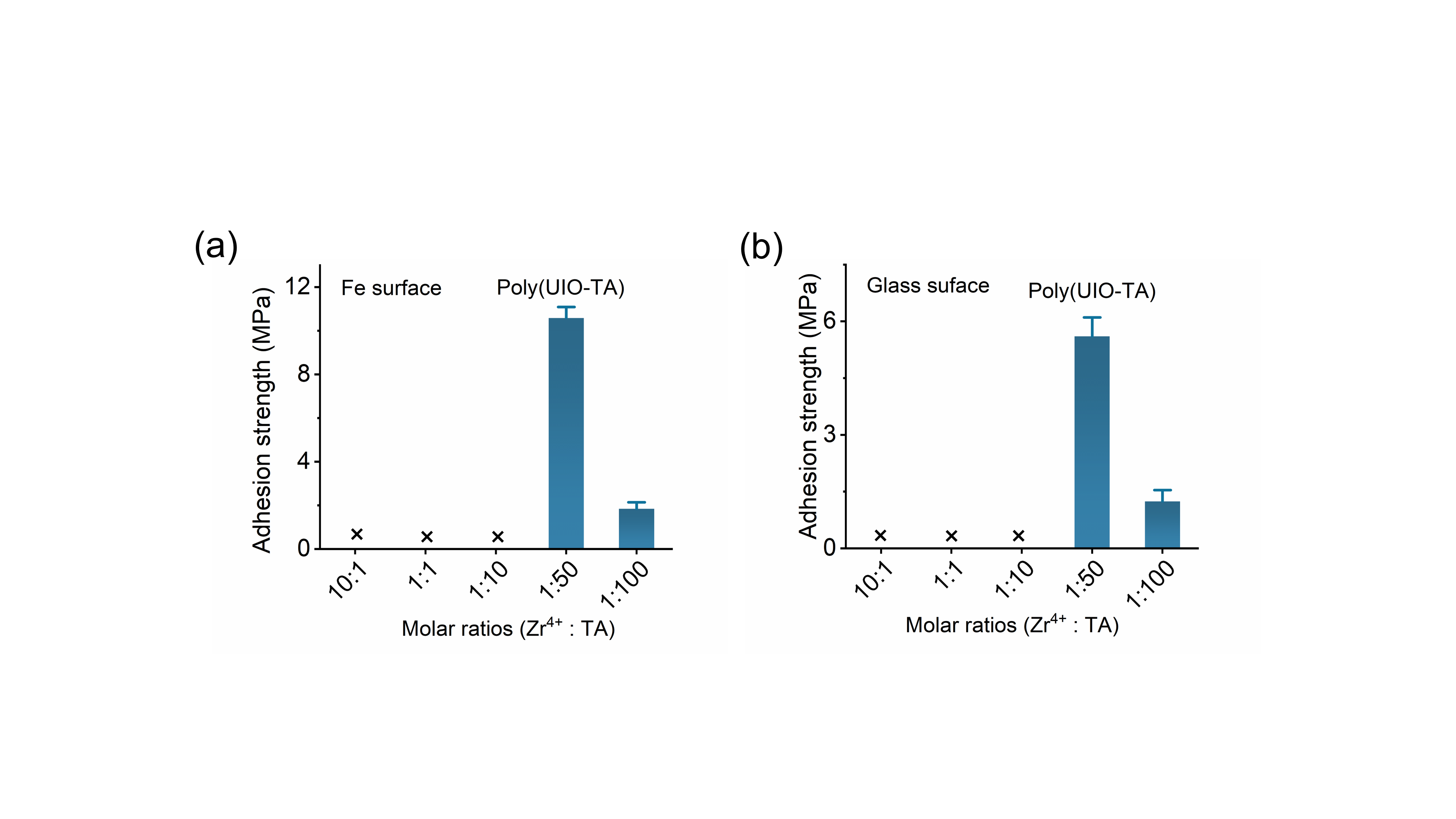


**Figure S19.** Adhesion strength of hybrid materials obtained from different molar ratio of Zr^4+^ to TA. (a) on steel surface; (b) on glass surface.

1. **Long-term adhesion strength of poly(UIO-TA)**


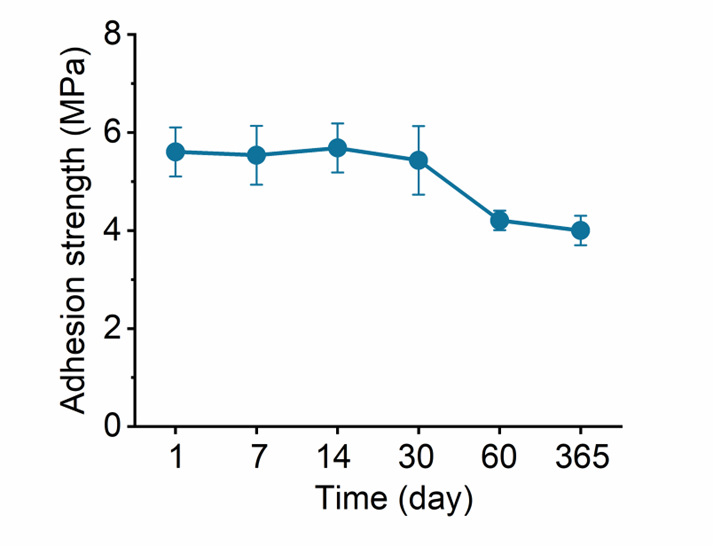


**Figure S20.** Time-dependent adhesion strength of poly(UIO-TA) on glass substrate.





**Figure S21.** Time-dependent adhesion strength of poly(UIO-TA) on a glass substrate under UV irradiation.

1. **Contact angle of poly(UIO-TA)**


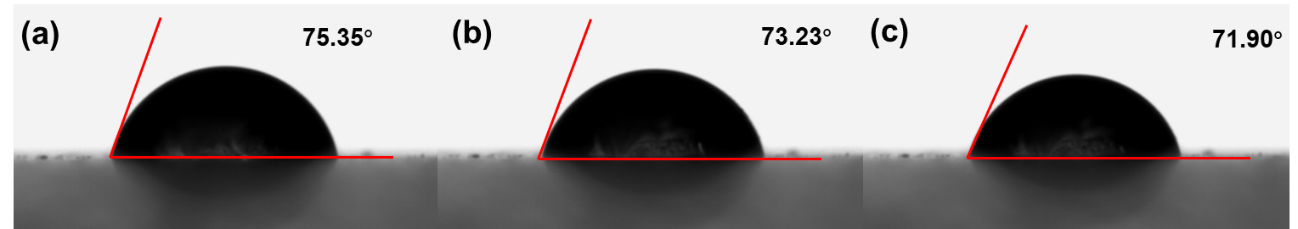


**Figure S22.** Contact angles of poly(UIO-TA) with water at 1 s (i), 30 s (ii) and 60 s (iii).

1. **X-ray photoelectron spectroscopy (XPS) spetra of poly(UIO-TA)**





**Figure S23.** Full scan XPS spectra of poly(UIO-TA) adhesion layer on steel substrate (a) before and (b) after treatment with NaOH solution.

1. **Solubility tests of poly(UIO-TA)**


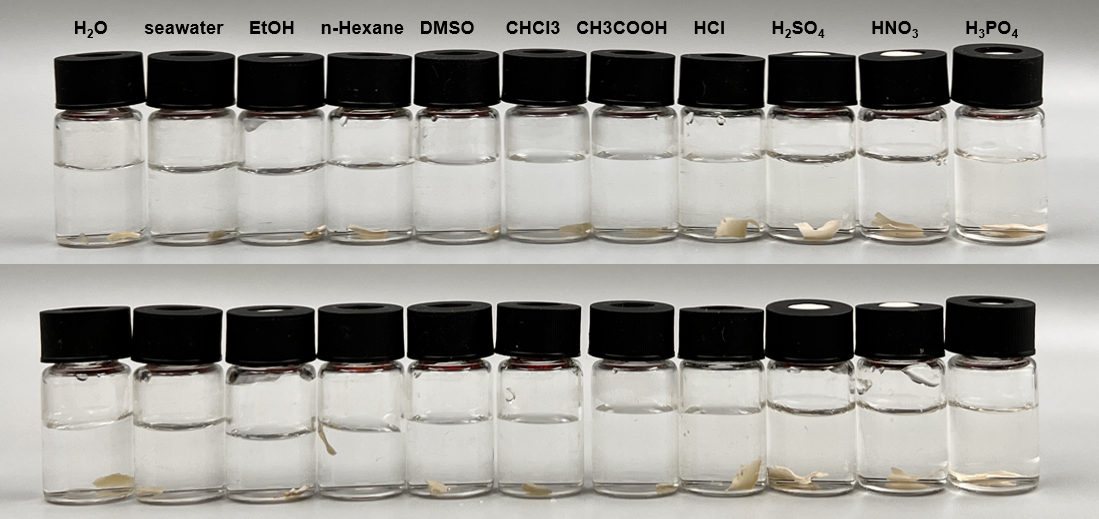


**Figure S24**. Solubility tests of poly(UIO-TA). Photographs of poly(UIO-TA) bulk immersed in various solvents and acidic solutions for 0 h (above) and 24 h (below).


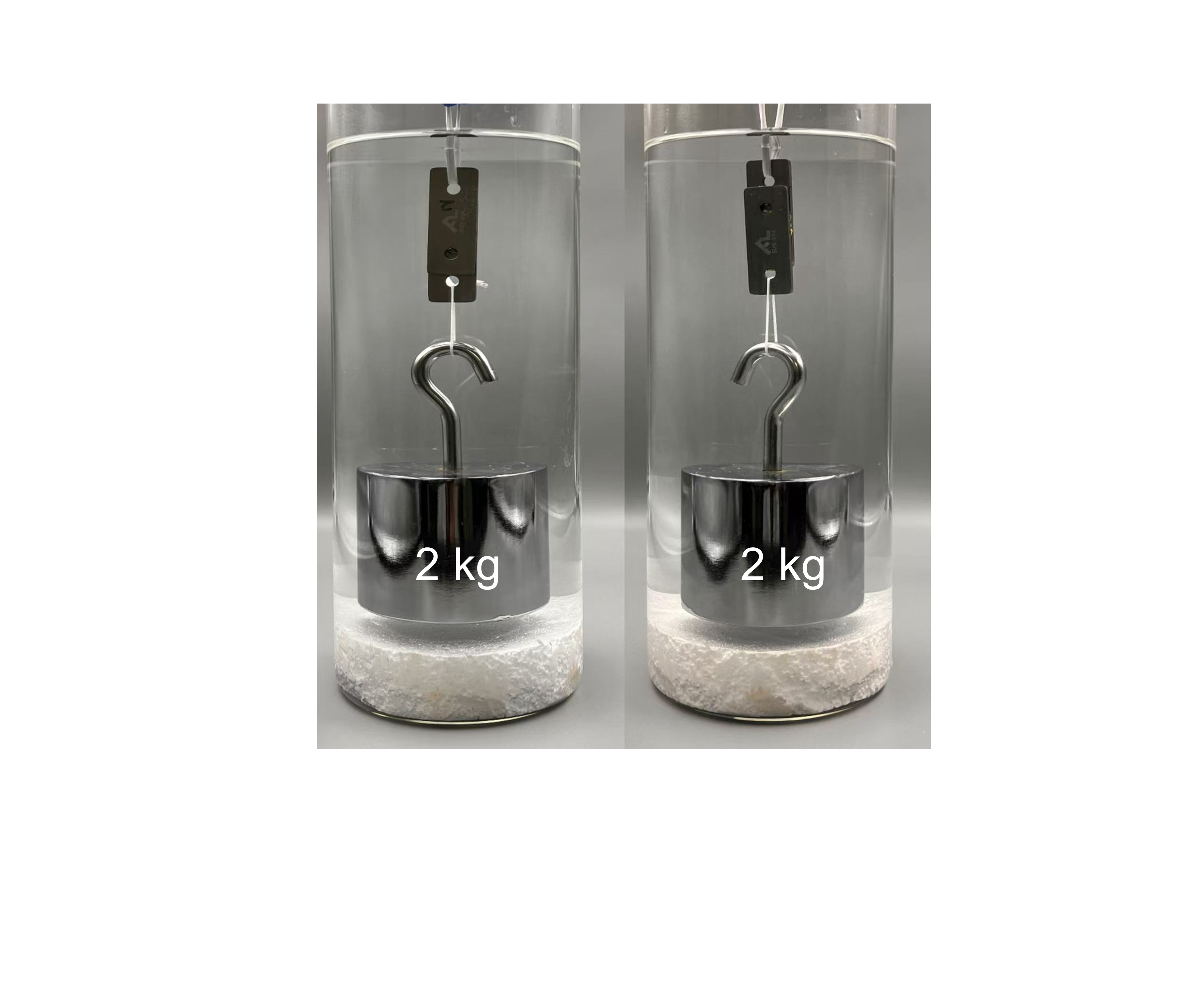


**Figure S25**. Macroscopic adhesion performance of poly(UIO-TA) in deionized water (left) and simulated seawater (right).





**Figure S26.** Adhesion strength of poly(UIO-TA) on glass surface after exposure to various strong acids for 24 h.


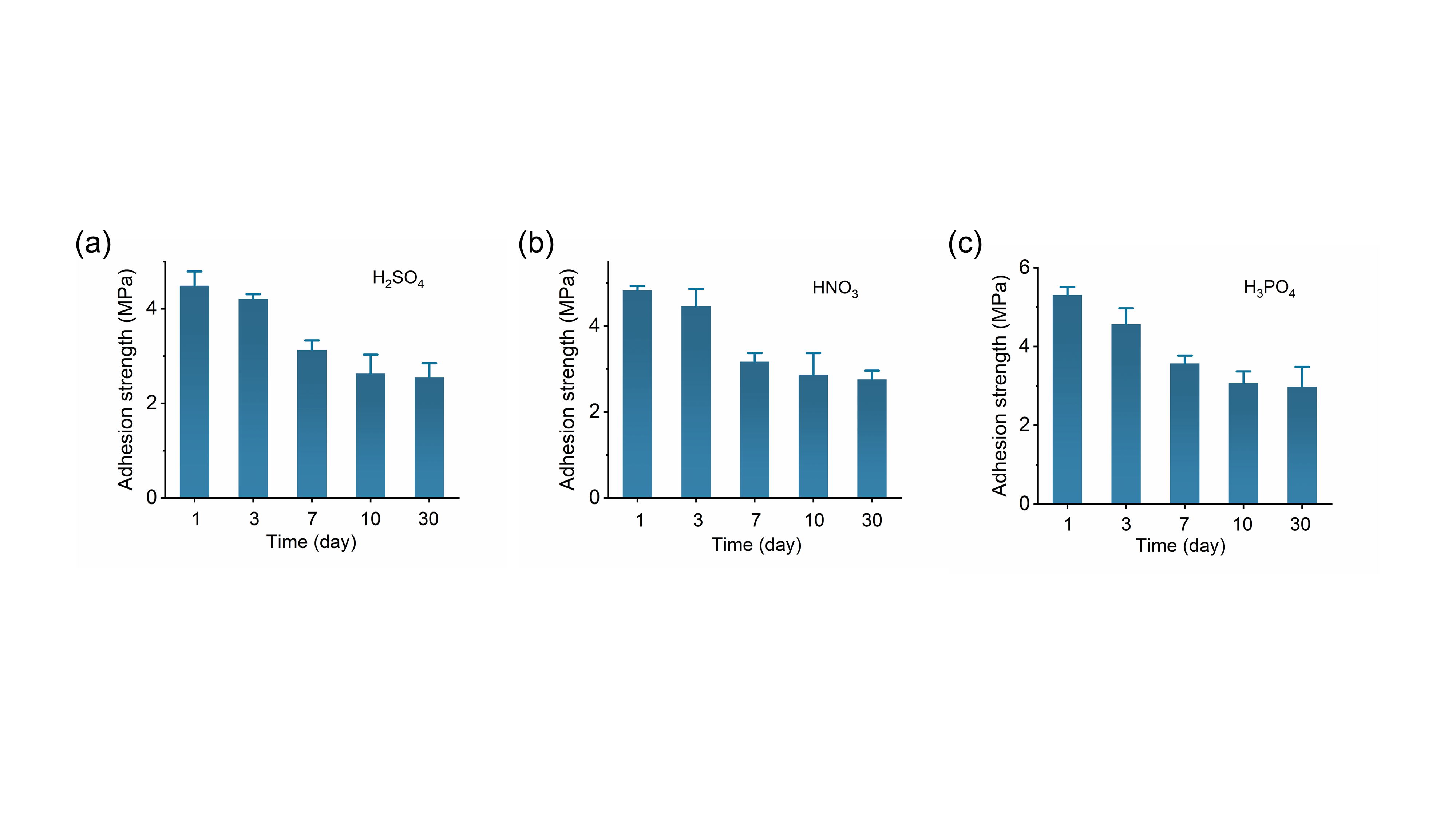


**Figure S27.** Time-dependent adhesion strength of poly(UIO-TA) with treatment of different strong acid solutions.


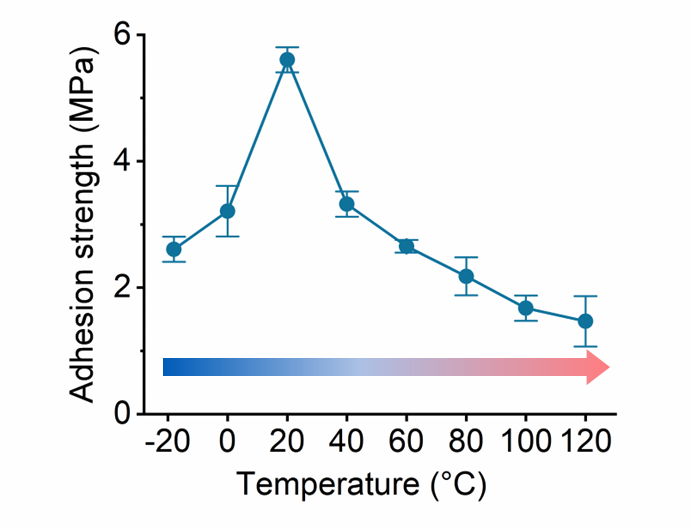


**Figure S28.** Temperature-dependent adhesion strength of poly(UIO-TA) on glass substrate.


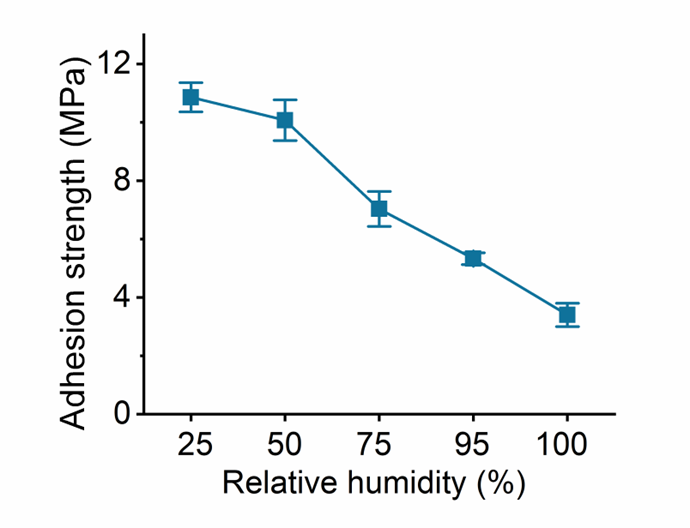


**Figure S29.** Adhesion strength of poly(UIO-TA) on steel at different relative humidity.





**Figure S30.** Radar plot of adhesion strength of poly(UIO-TA) and previously reported supramolecular adhesives under different conditions. The adhesion strength is measured in MPa.

The radar plot reveals that poly(UIO-TA) exhibits four unprecedented advantages. First, poly(UIO-TA) exhibits high adhesion strength of up to 4.64 MPa even at 120°C. Second, poly(UIO-TA) shows outstanding long-term adhesion performance. After 12 months, the adhesion strengths on steel and glass were measured at 6.34 MPa and 4.00 MPa, respectively, retaining 60%–70% of the initial adhesion strength. Third, poly(UIO-TA) displays distinct cyclic adhesion behavior. For example, the adhesion strength on steel increased from 4.59 MPa to 10.58 MPa during the first five cycles, then stabilized at approximately 10.58 MPa. In contrast, for most reversible adhesives, the adhesive strength remains constant or diminishes as the number of cycles increases. Finally, poly(UIO-TA) demonstrates exceptional resistance to a variety of solvents, including water, simulated seawater, organic solvents (e.g., dimethyl sulfoxide), and strong acid solutions (e.g., HCl, H_2_SO_4_, and HNO_3_). For instance, poly(UIO-TA) retained 2.11 MPa adhesion strength on steel after one month in DMSO and 2.51 MPa after one month in HCl solution, outperforming most reversible adhesives.

**Table S1. Comparison of the adhesion performance of poly(UIO-TA) and recently reported supermolecule adhesives.**

| **Name** | **Application Temperature**  **(**°C**)** | **Curing Condition** | **Adhesion strength (MPa)** | **Substrate** | **Ref.** |
| --- | --- | --- | --- | --- | --- |
| poly(TA-DIB-Fe) copolymer | 0  0 | 70~0 | ~2.7  ~1 | glass  teflon | 7 |
| PDMS-Pt-H | -20  100 | 120~RT | ~1.3  ~0.4 | steel | 8 |
| P1 | -18  70 | 52 | ~0.685  ~0.007 | steel | 9 |
| II-water | -18  70 | RT | ~0.69  ~0.21 | glass | 10 |
| PIL-2-TFSI | 10  65 | 100~RT | ~2.5  ~1.15 | glass | 11 |
| OE | 25  -80 | 60~RT | ~0.55  ~1.47 | steel | 12 |
| PDMS-HDI@MXeneZnO | 5  85 | NIR irradiation | ~2.253  ~0.1 | iron | 13 |
| **poly(UIO-TA)** | -18  120 | 100~RT | ~4.76  ~4.64 | steel | This work |

**Table S2**. **Comparison of the adhesion performance of poly(UIO-TA) and recently reported adhesive materials** **in different solvents.**

| **Name** | **Solvents** | **Time** | **Substrate** | Adhesion strength **(MPa)** | **Ref.** |
| --- | --- | --- | --- | --- | --- |
| poly(T*t*ADO-TA)-2 copolymer | seawater | 3 days | glass | ~0.6 | 14 |
| P1 | seawater | 12 months | glass | hanging 2 kg, no separation occurred | 8 |
| pDOPA-AD-MEA copolymer | KCl  NaCl MgCl_2_ ZnCl_2_ | / | silicon | ~4 kPa | 15 |
| SM/TA@PCD/ChNCs | 0.5 M NaCl | 24 h | wood | ~2.09 | 16 |
| Tri-HT | NaCl | 24 h | SS | ~4 | 19 |
| ZPIL | DMSO | 2 weeks | SS | ~1 | 17 |
| SP-DN | DMSO  HCl | 24 h | glass | ~0.8 | 18 |
| **poly(UIO-TA)** | seawater  DMSO  HCl | 30 days | steel | ~3.86  ~2.11  ~2.51 | This work |

**Table S3**. **Comparison of adhesion strength and cyclic number of poly(UIO-TA) and reported** **reversible adhesives.**

| **Name** | Cyclic number and **Substrate** | Adhesion strength **(MPa)** | **Ref.** |
| --- | --- | --- | --- |
| poly(T*t*ADO-TA)-2 copolymer | 10, glass | 4-5 | 14 |
| P2 | 10, steel  10, Al_2_O_3_ | 3-6  6-9 | 19 |
| poly(TA-DIB-Fe) copolymer | 30, steel | ~1.5 | 6 |
| PDMS-Pt-H | 100, steel | ~1.25 | 7 |
| P1 | 10, steel | ~4.17 | 8 |
| CAM | 5, mylar film | ~1 | 20 |
| II-water | 9, glass | ~1.88 | 9 |
| PIL-2-TFSI | 30, glass | ~4.44 | 10 |
| OE | 6, steel | ~0.94 | 11 |
| poly(A-C)s | 6, iron | ~5.18 (at 150℃) | 13 |
| Tri-HT | 10, SS | ~8 | 21 |
| **poly(UIO-TA)** | 7, steel  7, glass | ~10.5  ~5.6 | This work |

1. **References**

[1] J. Kansy, Microcomputer Program for Analysis of Positron Annihilation Lifetime Spectra. Nuclear Instruments and Methods in Physics Research Section A: Accelerators, Spectrometers, Detectors and Associated Equipment. *Nucl. Instrum. Meth. A* **1996**, *374*, 235–244.

[2] S. Grimme, Semiempirical GGA-type density functional constructed with a long-range dispersion correction. *J. Comput. Chem.* **2006**, *27*, 1787–1799.

[3] J. P. Perdew, Restoring the Density-Gradient Expansion for Exchange in a GGA for Solid and Surfaces. 2008 APS March Meeting. American Physical Society, **2008**.

[4] S. Grimme, J. Antony, S. Ehrlich, K. Helge, A Consistent and Accurate ab Initio Parametrization of Density Functional Dispersion Correction (DFT-D) for the 94 Elements H-Pu. *J. Chem. Phys.* **2010**, *132*, 154104.

[5] Y. Liu, W. A. I. Goddard, A Universal Damping Function for Empirical Dispersion Correction on Density Functional Theory. *Mater. Trans.* **2009**, *50*, 1664–1670.

[6] J. Moellmann, S. Grimme, DFT-D3 Study of Some Molecular Crystals. *J. Phys. Chem. C* **2014**, *118*, 7615–7621.

[7] Q. Zhang, C. Shi, D. Qu, Y. Long, B. L. Feringa, H. Tian, Exploring a Naturally Tailored Small Molecule for Stretchable, Self-healing, and Adhesive Supramolecular Polymers. *Sci. Adv.* **2018**, *4*, eaat8192.

[8] Z. Wang, K. Huang, X. Wan, M. Liu, Y. Chen, X. Shi, S. Wang, High-Strength Plus Reversible Supramolecular Adhesives Achieved by Regulating Intermolecular PtII···PtII Interactions. *Angew. Chem. Int. Ed.* **2022**, *61*, e202211495.

[9] X. Li, Y. Deng, J. Lai, G. Zhao, S. Dong, Tough, Long-Term, Water-Resistant, and Underwater Adhesion of Low-Molecular-Weight Supramolecular Adhesives. *J. Am. Chem. Soc.* **2020**, *142*, 5371−5379.

[10] Q. Zhang, T. Li, A. Duan, S. Dong, W. Zhao, P. J. Stang, Formation of a Supramolecular Polymeric Adhesive via Water−Participant Hydrogen Bond Formation. *J. Am. Chem. Soc.* **2019**, *141*, 8058−8063.

[11] J. [Zhang](https://advanced.onlinelibrary.wiley.com/authored-by/Zhang/Jun), Z. [Chen](https://advanced.onlinelibrary.wiley.com/authored-by/Chen/Zhanying), Y. [Zhang](https://advanced.onlinelibrary.wiley.com/authored-by/Zhang/Yan), S. [Dong](https://advanced.onlinelibrary.wiley.com/authored-by/Dong/Shengyi), Y. [Chen](https://advanced.onlinelibrary.wiley.com/authored-by/Chen/Yufang), S. [Zhang](https://advanced.onlinelibrary.wiley.com/authored-by/Zhang/Shiguo), Poly(ionic liquid)s Containing Alkoxy Chains and Bis(trifluoromethanesulfonyl)imide Anions as Highly Adhesive Materials. *Adv. Mater.* **2021**, *33*, 2100962.

[12] S. Wu, W. Wang, C. Cai, F. Li, S. Dong, Low-Molecular-Weight Supramolecular Adhesive with Resistance to Low Temperatures. *Chin. Chem. Lett.* **2023**, *34*, 107830.

[13] Y. Yan, L. Wei, J. Shao, X. Qiu, X. Zhang, X. Cui, J. Huang, S. Ge, A Near-Infrared Photothermal-Responsive Underwater Adhesive with Tough Adhesion and Antibacterial Properties. *Small* **2024**, *20*, 2310870.

[14] S. Yang, J. Bai, X. Sun, J. Zhang, Robust and Healable Poly(disulfides) Supramolecular Adhesives Enabled by Dynamic Covalent Adaptable Networks and Noncovalent Hydrogen-Bonding Interactions. *Chem. Eng. J.* **2023**, *461*, 142066.

[15] S. Wang, W. H. Hu, Y. Nakamura, N. Fujisawa, A. E. Herlyng, M. Ebara, M. Naito, Bio-Inspired Adhesive with Reset-On Demand, Reuse-Many (RORM) Modes. *Adv. Funct. Mater.* **2023**, *33*, 2215064.

[16] S. Chen, M. Bai, Q. Wang, X. Li, J. Shao, S. Q. Shi, W. Zhou, J. Cao, J. Li, A Strong and Tough Supramolecular Assembled β-Cyclodextrin and Chitin Nanocrystals Protein Adhesive: Synthesis, Characterization, Bonding Performance on Three-Layer Plywood. *Carbohydr. Polym.* **2024**, *333*, 121971.

[17] J. Zhang, H. Li, X. Zhou, Q. Hu, J. Chen, L. Tang, X. Yang, J. Gao, B. Liu, Y. Zhang, G. Zhao, S. Dong, S. Zhang, Adhesive Zwitterionic Poly(ionic liquid) with Unprecedented Organic Solvent Resistance. *Adv. Mater.* **2024**, *36*, 2403039.

[18] K. Wang, H. Wang, J. Li, Y. Liang, X. Xie, J. Liu, C. Gu, Y. Zhang, G. Zhang, C. Liu, Super-Stretchable and Extreme Temperaturetolerant Supramolecular-Polymer Double-Network Eutectogels with Ultrafast in Situ Adhesion and Flexible Electrochromic Behaviour. *Mater. Horiz.*, **2021**, *8*, 2520.

[19] K. Liu, P. Wu, Small Ionic-Liquid-Based Molecule Drives Strong Adhesives. *Angew. Chem. Int. Ed.* **2024**, *63*, e202403220.

[20] M. Kim, H. Lee, M. C. Krecker, D. Bukharina, D. Nepal, T. J. Bunning, V. V. Tsukruk, Switchable Photonic Bio-Adhesive Materials. *Adv. Mater.* **2021**, *33*, 2103674.

[21] J. Zhang, W. Wang, Y. Zhang, Q. Wei, F. Han, S. Dong, D. Liu, S. Zhang, Small-Molecule Ionic Liquid-Based Adhesive with Strong Room-Temperature Adhesion Promoted by Electrostatic Interaction. *Nat. Commun.* **2022**, *13*, 5214.
